# A Dense Brown Trout (*Salmo trutta*) Linkage Map Reveals Recent Chromosomal Rearrangements in the *Salmo* Genus and the Impact of Selection on Linked Neutral Diversity

**DOI:** 10.1534/g3.116.038497

**Published:** 2017-02-24

**Authors:** Maeva Leitwein, Bruno Guinand, Juliette Pouzadoux, Erick Desmarais, Patrick Berrebi, Pierre-Alexandre Gagnaire

**Affiliations:** * Institut des Sciences de l’Evolution, Unité Mixte de Recherche 5554 CNRS-IRD-EPHE, Université de Montpellier, 34095 Montpellier Cedex 5, France; † Département Biologie-Ecologie, Université de Montpellier, 34095 Montpellier Cedex 5, France

**Keywords:** linkage mapping, *Salmo trutta*, salmonids, RAD markers, chromosomal rearrangements, recombination rate

## Abstract

High-density linkage maps are valuable tools for conservation and eco-evolutionary issues. In salmonids, a complex rediploidization process consecutive to an ancient whole genome duplication event makes linkage maps of prime importance for investigating the evolutionary history of chromosome rearrangements. Here, we developed a high-density consensus linkage map for the brown trout (*Salmo trutta*), a socioeconomically important species heavily impacted by human activities. A total of 3977 ddRAD markers were mapped and ordered in 40 linkage groups using sex- and lineage-averaged recombination distances obtained from two family crosses. Performing map comparison between *S. trutta* and its sister species, *S. salar*, revealed extensive chromosomal rearrangements. Strikingly, all of the fusion and fission events that occurred after the *S. salar*/*S. trutta* speciation happened in the Atlantic salmon branch, whereas the brown trout remained closer to the ancestral chromosome structure. Using the strongly conserved synteny within chromosome arms, we aligned the brown trout linkage map to the Atlantic salmon genome sequence to estimate the local recombination rate in *S. trutta* at 3721 loci. A significant positive correlation between recombination rate and within-population nucleotide diversity (π) was found, indicating that selection constrains variation at linked neutral sites in brown trout. This new high-density linkage map provides a useful genomic resource for future aquaculture, conservation, and eco-evolutionary studies in brown trout.

A renewed interest for linkage mapping studies has recently occurred thanks to the simplified procedures for generating large genotype datasets in nonmodel organisms (Slate *et al.* 2008; Stapley *et al.* 2010; Ekblom and Galindo 2011). High-density linkage maps have been particularly developed in teleost fishes because of their high number of species, economic importance for aquaculture (*e.g.*, Sauvage *et al.* 2012; Zhao *et al.* 2013; Wang *et al.* 2015; Liu *et al.* 2016; Kumar and Kocour 2017), phylogenetic position within vertebrates (Amores *et al.* 2011; Rondeau *et al.* 2014; Wang *et al.* 2015; Kuang *et al.* 2016), and broad interests for eco-evolutionary and conservation/management issues (Everett *et al.* 2012; Gagnaire *et al.* 2013a; Kai *et al.* 2014).

The salmonid family, which has 11 genera and >60 species (Crête-Lafrenière *et al.* 2012), is a group of fish in which numerous initiatives have been developed to construct high-density linkage maps (Brieuc *et al.* 2014; Gonen *et al.* 2014; Limborg *et al.* 2015b; Larson *et al.* 2016; Tsai *et al.* 2016). Besides their applications for mapping traits of importance for aquaculture (Houston *et al.* 2012; Sauvage *et al.* 2012; Tsai *et al.* 2015), salmonid linkage maps have catalyzed molecular ecology and genome evolution studies over recent years. Several studies have used linkage map information for addressing eco-evolutionary and related conservation biology issues at the species level or among hybridizing taxa (Lamaze *et al.* 2012; Bourret *et al.* 2013; Perrier *et al.* 2013; Gagnaire *et al.* 2013b; Limborg *et al.* 2014; McKinney *et al.* 2015). A second category of studies have focused on understanding the evolutionary consequences of the whole genome duplication event (salmonid-specific fourth vertebrate whole genome duplication; WGD-Ss4R) that occurred within the salmonid family ∼60 MYA (Crête-Lafrenière *et al.* 2012), especially with regards to the partial rediploidization process, which is still underway and not totally understood yet (Berthelot *et al.* 2014; Brieuc *et al.* 2014; Kodama *et al.* 2014; Sutherland *et al.* 2016; Allendorf *et al.* 2015; Lien *et al.* 2016; Nugent *et al.* 2017). A significantly improved understanding of this process has been achieved through increased efforts to map and compare chromosomal rearrangements among species (inversions, translocations, fissions, and fusions), which have helped to reconstruct the evolutionary history of salmonid genome architecture (Kodama *et al.* 2014; Sutherland *et al.* 2016). Furthermore, linkage mapping approaches have also improved the characterization of the genomic regions that still exhibit tetrasomic inheritance patterns (Allendorf *et al.* 2015; Kodama *et al.* 2014; Lien *et al.* 2016; May and Delany 2015; Waples *et al.* 2016). The partial rediploidization of salmonid genomes is also known to challenge the development of genome-wide marker data sets. Indeed, duplicated loci that mostly reside in tetrasomic regions (Allendorf *et al.* 2015) are usually underrepresented or even excluded in salmonid genomic studies (Hohenlohe *et al.* 2011; Hecht *et al.* 2012; Gagnaire *et al.* 2013b; Larson *et al.* 2014; Perrier *et al.* 2013; Leitwein *et al.* 2016)

The Eurasian brown trout (*Salmo trutta* L. 1758), is one of the most widespread freshwater species in the Northern Hemisphere. It presents high levels of phenotypic diversity linked to its complex evolutionary history (Elliott 1994; Bernatchez and Osinov 1995; Bernatchez 2001). Natural brown trout populations have been intensely studied using molecular markers (*e.g.*, Hansen *et al.* 2000; Sanz *et al.* 2006; Thaulow *et al.* 2014; Berrebi 2015; Leitwein *et al.* 2016), but previous population genetics and phylogeography studies that usually relied on a limited number of markers did not integrate linkage map information (but see Hansen and Mensberg 2009; Hansen *et al.* 2010). The microsatellite linkage map available until now in brown trout (Gharbi *et al.* 2006) contains ∼300 markers (288 microsatellites and 13 allozymes), distributed over 37 of the 40 linkage groups (LGs) expected in this species (2*n* = 80, Martinez *et al.* 1991; Phillips and Ráb 2001). In order to follow the tendency toward an increasing number of markers in brown trout genetic studies (Leitwein *et al.* 2016), a higher density linkage map is needed to increase mapping resolution and provide positional information for genome scans and association studies. The development of a high-density linkage map in brown trout should find at least two direct applications.

First, although the brown trout is the closest relative of the Atlantic salmon (*S. salar* L. 1758), the two species drastically differ in their number of chromosomes (*S. salar* 2*n* = 54–58, Brenna-Hansen *et al.* 2012; *S. trutta* 2*n* = 80, Phillips and Ráb 2001), indicative of a high frequency of chromosome rearrangements. For understanding the chromosomal evolution in the *Salmo* genus, a comparison between the genome architecture of the Atlantic salmon (Lien *et al.* 2016) and the brown trout, using other salmonid linkage maps as outgroups, is needed. The availability of important genomic resources in salmonids coupled with recent methodological advances in linkage map comparisons (Sutherland *et al.* 2016) provide favorable conditions for new insights into the recent history of chromosomal rearrangement in the *Salmo* genus.

Second, another important application of a high-density linkage map in brown trout relates to understanding the consequences of human-mediated introductions of foreign and domestic genotypes in natural populations. The brown trout has been domesticated for decades, and wild populations have been heavily impacted by the stocking of genetically domesticated hatchery strains sometimes originating from evolutionary lineages distinct from the recipient populations (Elliott 1994; Laikre 1999; Berrebi *et al.* 2000; Bohling *et al.* 2016). These introductions have often resulted in the introgression of foreign alleles within wild brown trout populations, a generally negatively perceived phenomenon in conservation and management (Largiader and Scholl 1996; Poteaux *et al.* 1998, 1999; Hansen *et al.* 2000; Almodóvar *et al.* 2006; Sanz *et al.* 2006; Hansen and Mensberg 2009). However, the real evolutionary consequences of hybridization may take multiple facets (*e.g.*, outbreeding depression, heterosis, adaptive introgression, loss of adaptive variation), the individual effects of which remains largely unknown. In order to evaluate those potential fitness outcomes in brown trout, it would be necessary to combine the power of large SNP data sets (Leitwein *et al.* 2016) with a high-density linkage map and a detailed knowledge of chromosomal variation in recombination rate to perform comprehensive genome scans for introgression in natural populations.

The specific objectives of this study were: (i) to build a high-density linkage map in brown trout based on restriction site–associated DNA (RAD) markers using reciprocal crosses of individuals belonging to two distinct evolutionary lineages (Atlantic and Mediterranean; Bernatchez 2001); (ii) to characterize chromosome morphologies and centromere positions of metacentric chromosomes using the method recently developed in Limborg *et al.* (2015a); (iii) to perform map comparisons with the *S. salar* genome and an outgroup salmonid species to infer the timing of chromosome rearrangements during the recent evolutionary history of the *Salmo* genus; and (iv) to estimate the genome-wide local variation in recombination rate and assess the consequence of this variation on the genomic landscape of nucleotide diversity in brown trout.

## Materials and Methods

### Experimental crosses

Two F1 hybrid families, each comprising two parents and 150 offspring, were used for the linkage map analysis. Both were obtained by crossing one parent of Atlantic origin with one parent of Mediterranean origin (Bernatchez 2001). The F0 progenitors were either from a domestic Mediterranean strain lineage originally bred in the Babeau hatchery [described in Bohling *et al.* (2016)] and a domestic Atlantic strain lineage originating from the Cauterets hatchery and independently reared from the Mediterranean strain at the Babeau hatchery. The Cauterets strain is one of the common, internationally distributed Atlantic strains (comATL category; Bohling *et al.* 2016). Crosses were performed in December 2015 at the Babeau hatchery as follows: FAMILY 1, one Atlantic female × one Mediterranean male; and FAMILY 2, one Atlantic male × one Mediterranean female. Progenies were killed in February 2016, after full resorption of the yolk sac (fish size ∼2 cm).

### DNA extraction, library preparation, and sequencing

Whole genomic DNA was extracted from caudal fin clips for the four parents of the two crosses and from tail clips of 150 offspring of each family, using the commercial Thermo Scientific KingFisher Flex Cell and Tissue DNA kit. Individual DNA concentration was quantified using both a NanoDrop ND-8000 spectrophotometer (Thermo Fisher Scientific) and Qubit 1.0 Fluorometer (Invitrogen, Thermo Fisher Scientific). Double digest RAD sequencing library preparation was performed following Leitwein *et al.* (2016). Briefly, DNA was digested with two restriction enzymes (*Eco*RI-HF and *Msp*I) and the digested genomic DNA was then ligated to adapters and barcodes. Ligated samples with unique barcodes were pooled in equimolar proportions and CleanPCR beads (GC Biotech, Alphen aan den Rijn, The Netherlands) were used to select fragments sized between 200 and 700 bp. The size selected fragments were amplified by PCR and tagged with a unique index specifically designed for Illumina sequencing (Peterson *et al.* 2012). For progenies, PCR products were pooled in equimolar ratios into four pools. Each pool was sequenced in a single Illumina HiSequation 2500 (*i.e.*, 75 F1 progenies per lane), producing 125-bp paired-end reads. The four parents were sequenced together in a single lane in order to obtain a higher coverage depth.

### Genotyping pipeline

Bioinformatic analyses followed the same procedure as in Leitwein *et al.* (2016). Briefly, raw reads that passed an Illumina purity filter and were of sufficient FastQC quality (> 28) were demultiplexed, cleaned, and trimmed with process_radtags.pl implemented in Stacks v1.35 (Catchen *et al.* 2013). The Atlantic salmon genome (GenBank accession number: GCA_000233375.4_ICSASG_v2; Lien *et al.* 2016) was used to determine individual genotypes at RAD markers using a reference mapping approach. First, reads were aligned to the *S. salar* genome with the BWA-mem program (Li and Durbin 2010). Alignment result files were processed into pstacks to build loci and call haplotypes for each individual (using *m* = 3 and *α* = 0.05). Individuals with a minimum mean coverage depth under 7× were removed, resulting in a data set of 147 offspring in family 1 and 149 offspring in family 2. Hereafter, a RAD locus is defined as a sequence of 120 bp, and thus can contain more than one SNP defining different haplotypes. A reference catalog of RAD loci was built with cstacks from the four parents of the two crosses (family 1 and family 2). Each individual offspring and parent were then matched against the catalog with sstacks. Then, for each family independently, the genotypes module was executed to identify mappable makers that were genotyped in at least 130 offspring in each family, and with a minimum sequencing depth of five reads per allele. The correction option (-c) in the genotypes module was used for automated correction of false-negative heterozygote calls. The map type “cross CP” for heterogeneously heterozygous populations was selected to export haplotypic genotypes in the JoinMap 4.0 format (Van Ooijen 2006). For importation into JoinMap, only the loci shared between family 1 and family 2 were kept, leaving a total of 7680 mappable loci in both families. 

### Linkage mapping

Both family data sets were filtered into JoinMap 4.0 (Van Ooijen 2006) to remove (i) loci presenting with significant segregation distortion, (ii) loci in perfect linkage (*i.e.*, similarity of loci = 1), and (iii) individuals with high rates of missing genotypes (>25%). After this first filtering step, 5635 and 5031 loci remained in family 1 and family 2, respectively. Only those that were shared by the two families were kept, resulting in a subset of 4270 filtered informative loci. A random subset of 4000 loci common to both families were finally selected, which corresponds to the maximum number of loci handled by JoinMap 4.0. We then discarded four offspring in family 1 and six offspring in family 2 due to high rates of missing genotypes (>20%). Markers were then grouped independently within each family using the “independence LOD” parameter in JoinMap, with a minimum LOD value of 10. Unassigned markers were second assigned using the strongest cross-linkage option with a LOD threshold of 5. Marker ordering was finally estimated within each group using the regression mapping algorithm and three rounds of ordering. The Kosambi mapping function was used to estimate genetic distances between markers in centimorgan. Finally, loci with undetermined linkage were removed and a consensus map was produced between families using the map integration function after identifying homologous LGs between family 1 and family 2.

### Estimation of centromere location and chromosome type

The method recently developed by Limborg *et al.* (2015a) was used to identify chromosome types (acrocentric, metacentric) and to determine the approximate centromere position of metacentric chromosomes. This method uses phased progeny genotypes to detect individual recombination events in comparison with parental haplotype combinations. For each LG, the cumulated number of recombination events between the first marker and increasingly distant markers was computed from both extremities, using each terminal marker as a reference starting point. When covering a full chromosome arm, the recombination frequency (RFm) values are expected to reach a plateau of ∼0.5, thus allowing the determination of chromosome morphology and the approximate centromere location for metacentric chromosomes [see Limborg *et al.* (2015a) for details]. We used the subset of markers that were found heterozygous only in the female parent to phase progeny haplotypes within each family. We only focused on female informative sites because the lower recombination rate in male salmonids makes them less adequate for inferring centromere locations (Limborg *et al.* 2015a). Moreover, lower male recombination rates have been reported in brown trout (Gharbi *et al.* 2006). The phased genotypes data sets were used to estimate RFm for intervals between markers along each LG. Finally, we plotted the RFm calculated from each LG extremity to determine the chromosome type and the approximate location of the centromere for metacentric chromosomes.

### Map comparisons

The microsatellite linkage map established by Gharbi *et al.* (2006) was compared and anchored to our new high-density linkage map using the MAPCOMP pipeline (available at https://github.com/enormandeau/mapcomp/) (Sutherland *et al.* 2016). The microsatellite markers’ primer sequences retrieved from Gharbi *et al.* (2006), as well as the five microsatellites used in Leitwein *et al.* (2016) and the lactate dehydrogenase (*LDH-C1*) gene from McMeel *et al.* (2001) were aligned with BWA_aln (Li and Durbin 2010) to the Atlantic salmon reference genome. Only single hit markers with a quality score (MAPQ) above 10 were retained. The 3977 RAD markers integrated in the high-density *S. trutta* linkage map (see *Results*) were also aligned to the *S. salar* reference genome with BWA_mem. Microsatellite and LDH markers were anchored onto the RAD linkage map by identifying the closest RAD locus located on the same contig or scaffold. Pairing with closely linked RAD markers enabled us to position markers from the previous generation map onto the newly developed linkage map.

An additional comparison was performed between the *S. salar* high-density linkage map published by Tsai *et al.* (2016) and the *S. trutta* consensus linkage map developed in this study. We ran the MAPCOMP pipeline (Sutherland *et al.* 2016) to identify blocks of conserved synteny and orthologous chromosome arms between the Atlantic salmon and the brown trout maps, using the Atlantic salmon genome as a reference. Conserved synteny blocks were visualized with the web-based VGSC (Vector Graph toolkit of genome Synteny and Collinearity: http://bio.njfu.edu.cn/vgsc-web/). The brook charr (*Salvelinus fontinalis*) linkage map published in Sutherland *et al.* (2016) was used as an outgroup to identify chromosome rearrangements that occurred within the *Salmo* lineage.

In order to estimate the genome-wide averaged net nucleotide divergence between *S. trutta* and *S. salar* (*d_a_*), we used the mean nucleotide diversity within *S. trutta* (*π_w_*, averaged between Atlantic and Mediterranean lineages) (Leitwein *et al.* 2016), and the absolute nucleotide divergence between *S. trutta* and *S. salar* (*π_b_*, also called *d_xy_*). The net divergence was estimated as *d_a_* = *π_b_* − *π_w_*, by supposing that the mean nucleotide diversity in *S. salar* (which is not available) was close to the mean nucleotide diversity estimated in *S. trutta*. To estimate *π_b_*_,_ we used the 3977 consensus sequences of the RAD loci integrated in our *S. trutta* linkage map, and aligned them with BWA_mem to the Atlantic salmon reference genome. Then, the total number of mismatches divided by the total number of mapped nucleotides was used to provide an estimate of *π_b_*.

### Estimation of genome-wide recombination rates

To estimate local variation in recombination rate across the genome, we compared our newly constructed genetic map of *S. trutta* to the physical map of *S. salar* with MareyMap (Rezvoy *et al.* 2007). In order to reconstruct a collinear reference genome for each brown trout LG, physical positions of brown trout RAD markers on the salmon genome were extracted from the BWA_mem alignment for each block of conserved synteny previously identified with MAPCOMP. The Loess method was used to estimate local recombination rates. It performs a local polynomial regression to fit the relationship between the physical and the genetic distances. The window size, defined as the percentage of the total number of markers to take into account for fitting the local polynomial curve, was set to 0.9 to account for local uncertainties due to the high rate of chromosomal rearrangements between species. A recombination rate equal to the weighted mean recombination rate of the two closest markers on the genetic map was assigned to the markers that were not used for fitting.

### Correlation between recombination rate and nucleotide diversity

In order to test for a correlation between local recombination rate and nucleotide diversity in brown trout, we used the estimate of nucleotide diversity (*π*) in the Atlantic lineage (based on 20 individuals). These estimates of nucleotide diversity were taken from Leitwein *et al.* (2016), in which brown trout populations and hatchery strains of different origins showed highly correlated genome-wide variation patterns in nucleotide diversity. Markers with a recombination rate value >2 cM/Mb or *π* < 0.008 were considered as outliers and were therefore removed from the analysis. This resulted in a data set containing 3038 markers for which both recombination rate and nucleotide diversity information were available.

### Data availability

Supplemental Material, Table S1 contains RAD markers IDs, consensus sequences, mapping position on the brown trout linkage map, physical position on the Atlantic salmon genome, and estimation of local recombination rate. Table S2 contains correspondence between the microsatellite and the new RAD linkage map. Figure S1 shows RFm plots obtained for each female haploid data set. Figure S2 shows MapComp Oxford plot comparing the brown trout to the Atlantic salmon linkage map, with markers paired through the Atlantic salmon genome. Figure S3 shows distribution of female and male marker positions along each LG for FAMILY 1. File S1 is an archive containing the .loc and .map files from both families. Demultiplexed sequence reads aligned against the Atlantic salmon genome (in Bam file format) are available at NCBI Short Read Archive under accession number PRJNA371687.

## Results

### Construction of a high-density linkage map in S. trutta

On average, 35M of filtered paired-end reads were obtained for each of the four parents (two families), and 5M paired-end reads for each of the 300 F1 (150 progenies per family) analyzed in this study. The retained subset of 7680 informative markers common to both families had a mean individual sequencing depth of 112× for the parents and 15× for the progenies, ensuring a good genotype calling quality in both families. Among these, a subset of 4000 markers showing no segregation distortion in both families was retained to generate a consensus map. The resulting integrated map thus provides sex- and lineage-averaged genetic distances, which is an important feature considering the reported differences in recombination rates between male and female in salmonids (Allendorf *et al.* 2015; Tsai *et al.* 2015), and the existence of divergent geographic lineages in brown trout (Bernatchez 2001).

**Figure 1:**
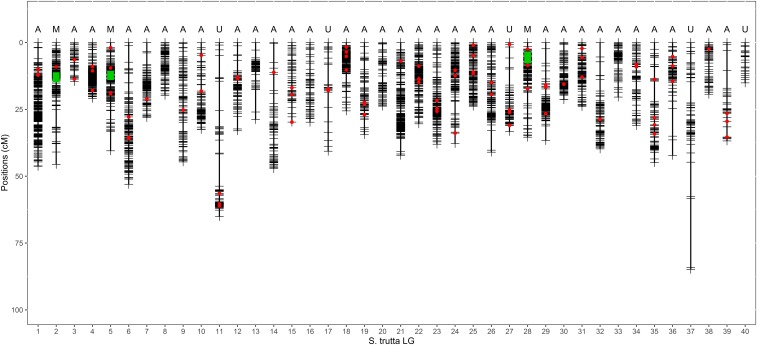
The brown trout (*Salmo trutta*) high-density linkage map. Horizontal black lines within each of the 40 LGs represent mapped RAD markers (*n* = 3977). The most probable chromosome type determined with the RFm method (Limborg *et al.* 2015a) is indicated above each LG. Green boxes indicate the approximate position of the centromere for metacentric LGs. Red dots represent the position of 89 additional markers that were transferred from the previous generation linkage map (see Table S2 for details). A, acrocentric; M, metacentric; U, undetermined.

A total of 3977 markers were assigned to 40 *S. trutta* (BT) LGs on the integrated map (Figure 1), corresponding to the expected number of chromosomes in this species (Phillips and Ráb 2001). The total map length was 1403 cM, and individual LGs ranged from 14.6 cM (BT-03; 47 markers) to 84.9 cM (BT-37; 63 markers). The number of markers per LG ranged from 23 for BT-40 (15.2 cM) to 252 for BT-01 (46.3 cM). Detailed information for each LG including the density of markers per centimorgan is reported in Table 1. Additional details concerning RAD markers IDs, consensus sequences, and mapping position on the salmon genome are reported in Table S1.

**Table 1:** Summary of the main features of the brown trout (*Salmo trutta*) high-density linkage map based on the 3977 mapped markers distributed over the 40 LGs described in this study

Brown Trout LG	Distance (cM)	Number of SNP Markers Mapped to Each LG	Density (markers/cM)	Recombination Rate (cM/Mb)	*LG_Map Gharbi*	Atlantic Salmon	Brook Charr	Northern Pike*^a^*
BT-01a	46.308	252	5.44	1.43	*BT-10*	***Ssa*16qb**	BC-05a	22.2
BT-01b					*BT-06*	*Ssa*29	BC-05b	22.1
BT-02a	45.621	130	2.85	0.82	*BT-05*	***Ssa*7q**	BC-02b	23.1
BT-02b					*BT-15*	S*sa*7p	BC-02a	24.1
BT-03	14.635	47	3.21	0.39	*BT-16*	***Ssa*3q**	BC-08a	11.2
					*BT-24*			
BT-04	20.902	249	11.91	0.4	*BT-32*	*Ssa*20qa	BC-40	13.2
BT-05b	40.51	140	3.46	0.56	*BT-6*	*Ssa*6q	BC-31	18.2
BT-05a					*BT-35*	***Ssa*6p**	BC-14	11.1
BT-06a	53.049	139	2.62	0.48	*BT-27*	**Ssa 12qa**	BC-03b	9.2
BT-06b						Ssa 12qb	BC-03a	17.1
BT-07	28.088	102	3.63	0.7	*BT-9*	Ssa 25	BC-24	16.2
BT-08	19.814	96	4.85	0.89	*-*	**Ssa 17qa**	BC-39	22.1
BT-09	44.687	88	1.97	1.18	*BT-13*	Ssa 1p	BC-12	15.2
BT-10	32.513	85	2.61	0.68	*BT-27*	Ssa 13qb	BC-08b	7.1
BT-11b	65.086	79	1.21	1.65	*BT-12*	**Ssa 5q**	BC-07a	20.1
BT-11a						Ssa 5p	BC-07b	4.2
BT-12	33.021	60	1.82	0.7	*-*	**Ssa 2p**	BC-29	20.2
BT-13	28.807	64	2.22	0.36	*-*	**Ssa 26**	BC-06a	2.1
BT-14	47.143	61	1.29	1.23	*BT-36*	Ssa 28	BC-27	5.2
BT-15	29.805	59	1.98	1.6	*BT-04*	**Ssa 4p**	BC-04b	25.1
						Ssa 23	BC-04a	8.1
BT-16	29.837	56	1.88	0.59	*-*	Ssa 1qb	BC-13	14.1
BT-17	40.655	24	0.59	1.13	*BT-28*	Ssa 15qb	BC-30	12.2
BT-18	25.58	207	8.09	0.5	*BT-16*	Ssa 3p	BC-11	3.2
BT-19	34.485	69	2	1.04	*BT-22*	Ssa 24	BC-06b	13.1
BT-20	23.814	60	2.52	0.65	*-*	**Ssa 2q**	BC-42	9.1
BT-21	42.194	187	4.43	1.43	*BT-25*	Ssa 16qa	BC-20	19.2
BT-22	30.433	145	4.76	0.6	*BT-30*	Ssa 4q	BC-09	7.2
BT-23b	38.057	141	3.7	0.5	*BT-02*	Ssa 9qb	BC-33	4.1
BT-23a						**Ssa 9qc**	BC-38	1.2
BT-24	37.805	139	3.68	1.11	*BT-31*	Ssa 21	BC-26	16.1
BT-25	23.843	131	5.49	0.63	*BT-07*	Ssa 10qb	BC-15	19.1
BT-26	41.025	113	2.75	0.45	*BT-33*	Ssa 22	BC-21	17.2
BT-27	33.275	111	3.34	0.21	*BT-26*	Ssa 13qa	BC-18	12.1
BT-28	35.425	109	3.08	1.05	*BT-23*	Ssa 18qa	BC-36	6.2
						Ssa 18qb	BC-32	24.1
BT-29	36.675	105	2.86	0.52	*BT-29*	Ssa 27	BC-23	10.1
BT-30	21.4	102	4.77	0.37	*BT-23*	Ssa 1qa	BC-01b	6.1
BT-31	24	99	4.13	1.33	*BT-02*	Ssa 9qa	BC-35	15.1
BT-32	39.782	87	2.19	0.81	*-*	Ssa 14qa	BC-22	3.1
BT-33	20.438	73	3.57	1.23	*-*	**Ssa 8q**	BC-41	25.2
BT-34	31.085	67	2.16	1.03	*BT-14*	**Ssa 20qb**	BC-25	1.1
BT-35	44.948	68	1.51	1.22	*BT-12*	Ssa 10qa	BC-17	8.2
					*BT-35*			
BT-36a	42.241	61	1.44	0.44	*BT-13*	**Ssa 11qa**	BC-28	2.2
BT-36b						Ssa 11qb	BC-10	14.2
BT-37	84.844	63	0.74	4.1	*-*	Ssa 14qb	BC-34	10.2
BT-38	19.365	49	2.53	1.57	*BT-15*	**Ssa 17qb**	BC-37	23.2
BT-39	36.825	37	1	1.08	*BT-28*	Ssa 15qa	BC-19	18.1
BT-40a	15.12	23	1.52	0.58	*-*	Ssa 19qb	BC-01a	5.2
BT-40b						Ssa 19qa	BC-16	21.2
Whole Genome	1403.14	3977	3.15	0.88				

The correspondence with LGs of the former generation linkage map established by Gharbi *et al.* (2006) is reported. The mean recombination rate is reported for each LG, as well as syntenic relationships with homologous chromosome arms from Atlantic salmon (*S. salar*), the brook charr (*Salvelinus fontinalis*) used as an outgroup for the *Salmo* genus, and the northern pike (*Esox lucius*) used as a nonduplicated outgroup for salmonids. The northern pike chromosomes are listed twice to accommodate the duplicate orthologs in the salmonid species (Sutherland *et al.* 2016). Boldface corresponds to the seven pairs involved in homeologous pairing (*i.e.*, tetrasomic inheritance) in *S. salar* (Lien *et al.* 2016).

a Data retrieved from Table 2 of Sutherland *et al.* (2016).

### Chromosome morphology and centromere location

**Figure 2:**
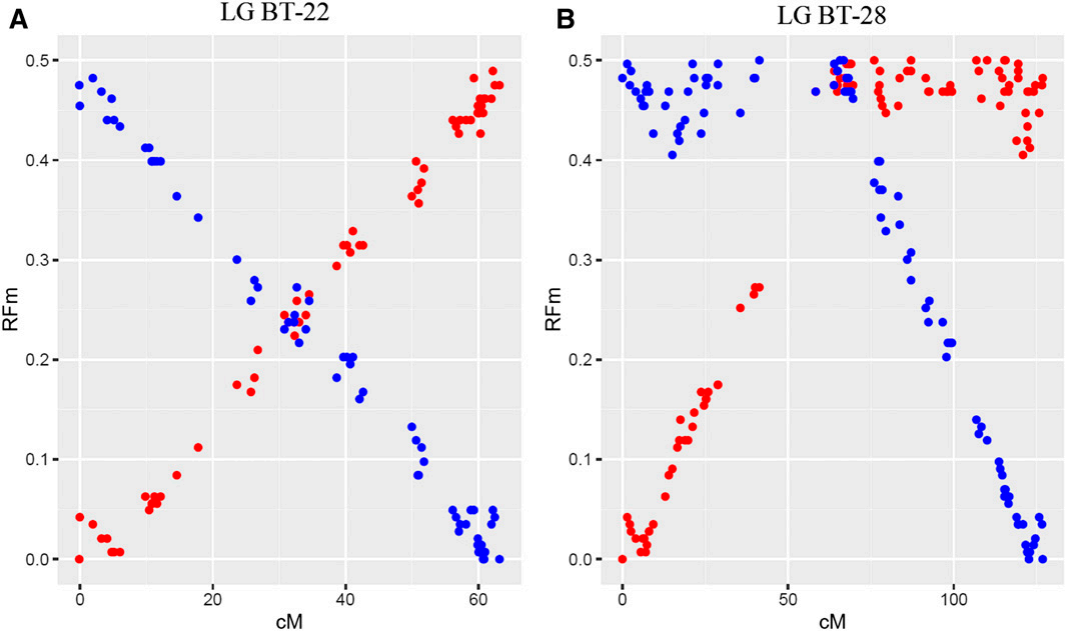
Plots illustrating the recombination frequency estimates (RFm) for intervals between markers along two different LGs. For each LG, RFm was calculated from both chromosomal extremities (right: red circles; left: blue circles), using each of the two terminal markers as a reference starting point. The RFm plot of (A) LG BT-22 illustrates a classical acrocentric pattern, the centromere position remains undetermined with regard to which LG extremity, while (B) LG BT-28 displays a classical metacentric pattern with a centromere position ∼70 cM. The RFm plots of all brown trout LGs obtained in the two families are illustrated in Figure S1.

Individual LG RFm plots obtained using estimates of recombination frequencies in progeny were generally similar between family 1 and family 2 (Figure S1). Two examples of RFm plots obtained in family 1 are reported in Figure 2 for BT-22 and BT-28. Following Limborg *et al.* (2015a), the pattern observed for BT-22 typically reflects an acrocentric chromosome type (paired straight lines; Figure 2A), whereas BT-28 illustrates a metacentric pattern (mirrored hockey stick shapes; Figure 2B) with the centromere located at ∼70 cM. The total number of probable acrocentric chromosomes (Figure 1; type A, *n* = 32) largely exceeded the number of metacentric chromosomes (Figure 1; type M, *n* = 3). The approximate position of the centromere was determined for each of the three metacentric LGs (Figure 1; green boxes). The chromosome type remained undetermined for five LGs (Figure 1; type U) due to low resolution of the RFm plots (Figure S1).

### Linkage map comparisons

A total of 87 microsatellite markers [82 out of 160 from Gharbi *et al.* (2006), five out of five from Leitwein *et al.* (2016)] and the LDH locus (McMeel *et al.* 2001) were successfully mapped to the Atlantic salmon genome, and anchored to the new brown trout linkage map using their pairing with RAD markers, following the method developed in Sutherland *et al.* (2016) (Figure 1; red dots). The correspondence between the microsatellite linkage map and the new RAD linkage map is provided in Table S2.

**Figure 3:**
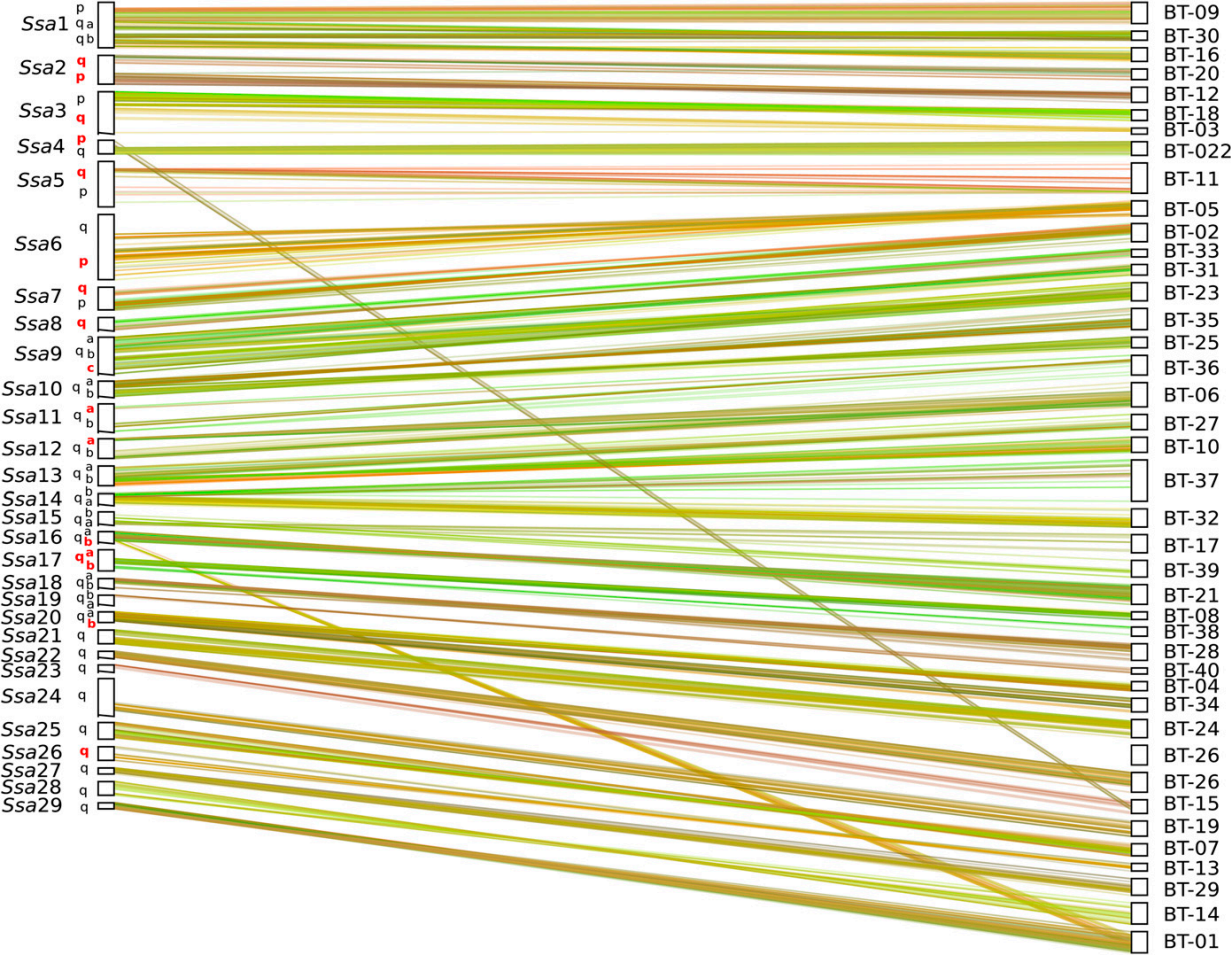
Dual synteny plot showing conserved syntenic blocks and chromosome rearrangements between *Salmo salar* (*Ssa*) and *Salmo trutta* (BT) LGs. Chromosomes arms (p, q_a_ and q_b_) are specified for *S. salar* LGs, and the seven pairs of chromosome arms that still exhibit residual tetrasomy in salmon appear in red (Lien *et al.* 2016).

The identification of orthologous chromosomes arms between S. *trutta* (40 LGs) and *S. salar* (29 LGs) revealed numerous differences, including fusions and fissions between the two species (Figure 3 and Table 1). We found 15 one-to-one ortholog pairs (*i.e.*, one brown trout LG corresponds to a single Atlantic salmon LG, as for example *Ssa*6 and BT-5, or *Ssa*28 and BT-14; Figure 3 and Table 1), 12 cases where Atlantic salmon chromosomes correspond to either two or three brown trout LGs (*e.g.*, *Ssa*1, *Ssa*2; Figure 3), and only two cases where brown trout LGs correspond to two Atlantic salmon chromosomes (BT-1 and BT-15; Figure 3). Between-species intrachromosomal inversions were also observed, as for example an inversion flanked by noninverted regions between *S. salar Ssa*6 and *S. trutta* BT-5 (Figure S2). Apart from these inter- and intrachromosomal rearrangements, a strongly conserved synteny was observed between the brown trout and the Atlantic salmon genomes. We estimated the average net nucleotide divergence between *S. trutta* and *S. salar* to *d_a_* = 0.0187 (with *π_b_* = 0.0228, and *π_w_* = 0.0041).

**Figure 4:**
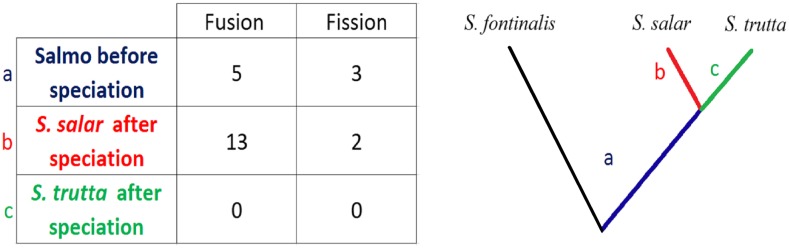
Summary of fission and fusion events within the *Salmo* genus, that were oriented using the brook charr (*Salvelinus fontinalis*) as an outgroup species. Rearrangements are classified with regards to their occurrence (a) before the *S. salar*/*S. trutta* speciation, (b) in the *S. salar* branch after speciation, or (c) in the *S. trutta* branch after speciation.

The identification of orthologous genomic regions between the brook charr, the brown trout, and the Atlantic salmon (Table 1) allowed inference of ancestral and derived chromosomes structures in the *Salmo* genus. We found eight chromosomal rearrangements that occurred in the ancestral lineage of the *Salmo* genus before speciation between *S. trutta* and *S. salar*. This includes five fusion events and three fission events (Figure 4 and Table 1). While no species–specific rearrangement was detected in the *S. trutta* branch, the Atlantic salmon branch contained two fission events and 13 fusion events (Figure 4). Among these fusions, the one between BT-17 and BT-39 corresponds to *Ssa*15, which is known to contain the salmonid sex-determining gene *sdY* in the Atlantic salmon (Li *et al.* 2011; Yano *et al.* 2013). Since the microsatellite locus *OmyRT5TUF* was consistently shown to be linked to *sdY* in the brown trout (Gharbi *et al.* 2006; Li *et al.* 2011), our results indicate that LG BT-39 might contain the sex-determining gene in brown trout (Table S2), which will need to be investigated further.

### Estimations of genome-wide and local recombination rates

**Figure 5:**
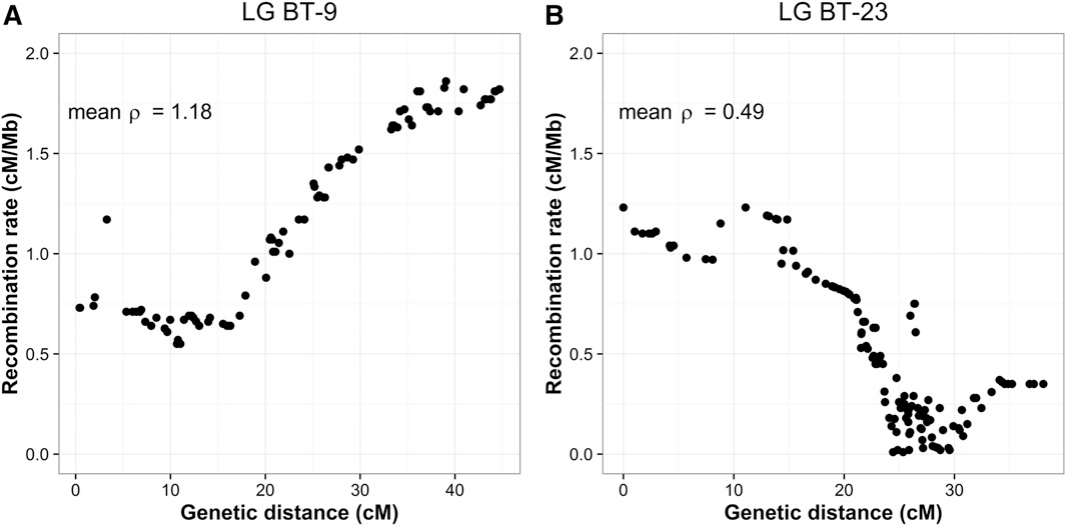
Estimates of local recombination rate (*ρ*) in centimorgan per megabase for markers located along two *Salmo trutta* linkage groups: (A) LG BT-9 and (B) LG BT-23. The average recombination rate (mean *ρ*) for each LG is given at the top left side of each panel. Local recombination rate estimates for other *S. trutta* LGs are reported in Table S1 and the average recombination rate of each LG is provided in Table 1.

**Figure 6:**
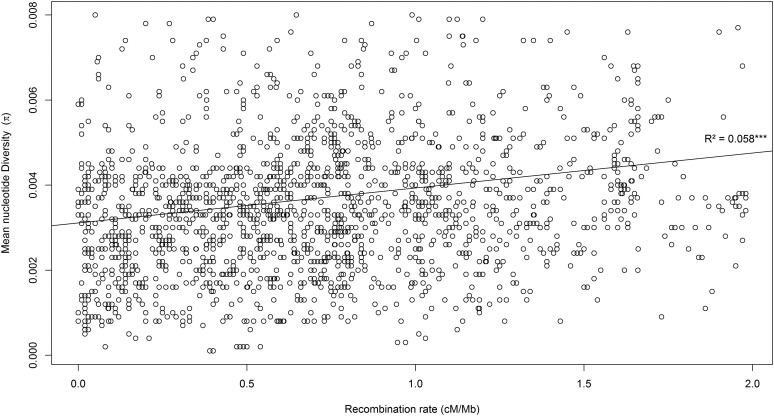
Genome-wide positive correlation between the mean nucleotide diversity in the Atlantic brown trout lineage (*π*) and the local recombination rate (*ρ*, in centimorgan per megabase), based on 3038 SNP markers (*R*^2^ = 0.058; *p*-value < 0.001).

The brown trout genome-wide recombination rate averaged across the 3721 markers anchored on the Atlantic salmon reference genome (RAD markers mapped to ungrouped scaffolds were discarded) was estimated at 0.88 ± 0.55 cM/Mb, and ranged from 0.21 cM/Mb (BT-27) to 4.10 (BT-37) across LGs (Table 1 and Table S1). Chromosomal variation in local recombination rate was observed for most chromosomes (*e.g.*, BT-9 and BT-23, Figure 5). A significant positive correlation between local recombination rate and population nucleotide diversity was detected (*R*^2^ = 0.058; *p*-value < 0.001; Figure 6).

## Discussion

This study describes the first high-density linkage map in the brown trout *S. trutta*, which extends the microsatellite linkage map already available in this species (Gharbi *et al.* 2006). The map covers 1403 cM and encompasses 3977 nondistorted markers distributed across 40 LGs, the centromere location of which was determined for 35 LGs. Results showed consistent chromosome number and morphologies compared to species karyotype (2*n* = 80, NF = 100 chromosome arms; Phillips and Ráb 2001). High-resolution mapping (average density of 3.15 markers per cM) was insured by the use of two mapping families, each containing 150 offspring, and the number of informative markers in both families was close to the limit over which saturation is expected (*i.e.*, 4200 markers). The homogeneous distribution of makers across the genome, and the estimation of averaged recombination distances between both evolutionary lineages and sexes provide a highly valuable resource for future genomic, aquaculture, conservation, and eco-evolutionary studies in brown trout. Beyond these general interests, this new linkage map also reveals strikingly different rates of chromosomal rearrangements that happened between the brown trout and the Atlantic salmon since their recent divergence.

### A new generation RAD linkage map in brown trout

Comparison between the newly developed linkage map and the previous microsatellite map was made possible by the integration of 82 microsatellite markers from the former generation map (Gharbi *et al.* 2006). This enabled us to identify the correspondence between homologous LGs from the two maps, and to detect cases of LG merging (eight out of 40) and LG splitting (nine out of 40) in the microsatellite map compared to the RAD linkage map. Consistent with the reported overrepresentation of acrocentric compared with metacentric chromosomes in the brook charr (*i.e.*, eight metacentric and 34 acrocentric, Sutherland *et al.* 2016), we found three metacentric and 32 acrocentric chromosomes in the brown trout. The morphology of five chromosomes could not be determined from the analysis of RFm plots alone. However, BT-11 was likely a metacentric chromosome based on its correspondence with the Atlantic salmon LG *Ssa*5 (Figure 3), and BT-17, BT-27, and BT-37 were likely acrocentric chromosomes due to single correspondences with acrocentric chromosomes of the Atlantic salmon and brook charr linkage maps (Table 1). This represents a total number of chromosome arms ranging from 88 to 90, which is lower but quite close to the 96–104 interval previously estimated in *S. trutta* (Martinez *et al.* 1991; Phillips and Ráb 2001). Our assessment of chromosome type did not perfectly match the results from Gharbi *et al.* (2006), since the three metacentric chromosomes identified here were found to be either acrocentric or undetermined in their study. However, the metacentric chromosomes BT-2 and BT-5 identified here had more than a single matching LG in the former microsatellite map, which suggests that these homologs represent the two telomeric regions of a larger metacentric LG that has been reconstructed in our study. On the contrary, the three metacentric chromosomes identified in Gharbi *et al.* (2006) appeared to be acrocentric using the phase information available from our data set. This could be the consequence of pseudolinkage in the microsatellite map built using males of hybrid origin, in which the preferential pairing of conspecific homeologs followed by alternate disjunction may have generated an excess of nonparental gametes (Morrison 1970; Wright *et al.* 1983; Allendorf *et al.* 2015). In our study, the possible effect of pseudolinkage was avoided by using nonhybrid parents with a single lineage ancestry, thus possibly explaining the observed differences with the previous generation map.

Besides pseudolinkage, another broader consequence of past whole genome duplication in salmonids is the existence of duplicated loci that share the same alleles due to homeologous recombination in telomeric regions (Wright *et al.* 1983; Allendorf and Danzmann 1997; Allendorf *et al.* 2015). These residual tetrasomic regions challenge traditional linkage map reconstruction methods since duplicated loci (isoloci) often show Mendelian segregation distortion, and are therefore frequently excluded from linkage mapping analyses. Alternative approaches can be used to detect homeologous regions in experimental crosses (Lien *et al.* 2011; Brieuc *et al.* 2014; Waples *et al.* 2016). In brown trout, the location of duplicated regions have been preliminarily established from genome-wide patterns of RAD markers density and nucleotide diversity projected on the Atlantic salmon reference genome (Leitwein *et al.* 2016). Although our new RAD linkage map is based on nondistorted markers and therefore probably displays a reduced marker density in regions exhibiting tetrasomic inheritance, it does not completely exclude such regions. This is illustrated by the mapping of multiple RAD loci within the telomeric region of the seven pairs of homeolog chromosome arms that retain residual tetrasomy in the Atlantic salmon genome (Lien *et al.* 2016) (Figure 3). Except for three of these 14 regions that received relatively few markers (*Ssa*3q, *Ssa*11qa, and *Ssa*17qa on the salmon map; Figure 3), the remaining arms were well covered in our RAD linkage map, indicating that the filtering of non-Mendelian markers did not completely exclude the telomeric regions affected by homeologous crossovers. This feature of our RAD linkage map should insure a more even recombination rate estimation between sexes (Lien *et al.* 2011), since the commonly observed lower recombination rate in males compared to females in salmonids could be partly due to a poor characterization of telomeric regions in mapping studies (Allendorf *et al.* 2015). In order to evaluate the extent to which male and female informative markers tend to map to distinct regions, we compared their positions along each LG in family 1 that received the highest number of mapped markers (Figure S3). We observed that on average, informative markers in the male parent were less evenly distributed across LGs and tended to aggregate in regions containing lower densities of female informative markers. Although this might also reflect a poor positioning of male markers due to low recombination rates in males (Sutherland *et al.* 2016), the sex-averaged recombination distances in our consensus linkage map should provide relevant estimates of recombination rate along chromosomes for future eco-evolutionary studies. For instance, understanding genome-wide introgression patterns in nature needs to integrate information from both male and female recombination rate.

Following the same idea, integrating maps between parents of different lineage origins (Atlantic and Mediterranean) allowed us to generate a consensus linkage map in which the potential differences in recombination rates between these two allopatric lineages are smoothened. Our results suggest that no fusion polymorphism occurs between the two brown trout lineages. This is consistent with the lack of species–specific chromosomal rearrangement following speciation with the Atlantic salmon (Figure 4), and the stable chromosome number previously observed in brown trout (Hartley and Horne 1984; Martinez *et al.* 1991; Phillips and Ráb 2001). Therefore, the lineage-averaged recombination distances provided here are probably not affected by potential interchromosomal rearrangements within the *S. trutta* species. The lineage-averaged consensus map will therefore be valuable to future conservation genomic studies, considering the extensive human-mediated admixture between Atlantic and Mediterranean brown trout populations in southern Europe (Berrebi *et al.* 2000; Sanz *et al.* 2006; Thaulow *et al.* 2014; Berrebi 2015).

### The recent evolution of chromosomal rearrangements in the Salmo genus

Salmonids have been undergoing a rediploidization process since a whole genome duplication event that occurred ∼60 MYA (Crête-Lafrenière *et al.* 2012; Near *et al.* 2012; Macqueen and Johnston 2014; Lien *et al.* 2016). This process, also described as functional diploidization by Ohno (1970), was accompanied by numerous chromosomal rearrangements within the salmonid family, making linkage maps of prime importance for studying the complex history of chromosomal evolution in salmonids (Sutherland *et al.* 2016). Several studies have pointed out a particularly high number of chromosomal rearrangements in the lineage leading to the Atlantic salmon, involving mainly chromosome fusions that reduced the number of chromosomes to 29 in this species (Allendorf and Thorgaard 1984; Lien *et al.* 2016; Sutherland *et al.* 2016). However, the timing of these rearrangements remains largely unknown due to the lack of a closely related species in previous studies. Because *S. trutta* is the most closely related species to *S. salar* among salmonids, the new brown trout RAD linkage map allowed for inferring the phylogenetic positions of the chromosome arm rearrangements that occurred within the *Salmo* genus. The ancestral state of *Salmo* chromosome structure was further resolved using the brook charr as an outgroup within salmonids, as well as other salmonid species (*e.g.*, the lake whitefish *Coregonus clupeaformis*) when necessary (Sutherland *et al.* 2016). This enabled us to identify five fusion events and three fission events (Table 1) that occurred in the *Salmo* ancestral lineage before the Atlantic salmon/brown trout speciation. This is relatively small compared to the total number of chromosomal rearrangements that occurred within the whole *Salmo* branch. Thus, most of the fission and fusion events that were observed in the Atlantic salmon genome (Sutherland *et al.* 2016) most probably occurred after the divergence between the two *Salmo* species. The net nucleotide divergence between *S. salar* and *S. trutta* was here estimated to 1.87%, which indicates a relatively recent divergence time between these two sister species. Surprisingly, all of the interchromosomal rearrangements that occurred after the salmon/trout speciation happened in *S. salar*. Indeed, we did not observe any fusion/fission events within the *S. trutta* branch (Figure 4), whereas 15 rearrangements mostly involving fusions (13 fusions and two fissions) specifically occurred in the Atlantic salmon branch. These Robertsonian translocations explain the increased number of metacentric chromosomes in the Atlantic salmon compared to the brown trout, which were produced by the recent fusion of acrocentric chromosomes (*Ssa*1 to *Ssa*4; Figure 3 and Table 1). However, some fusion events have also likely resulted in the formation of acrocentric chromosomes in the Atlantic salmon (*e.g.*, *Ssa*15; Figure 3 and Table 1), which could partly explain the apparent loss of chromosomes arms in this species (Allendorf and Thorgaard 1984). Finally, intrachromosomic rearrangements also occurred along the *S. salar* branch, as illustrated by the inversion detected within *Ssa*6, the homologous LG to BT-5 (Figure 3 and Figure S2). Altogether, our results support the previous views that many chromosomal rearrangements have occurred within the *Salmo* lineage (Allendorf and Thorgaard 1984; Sutherland *et al.* 2016), but also demonstrate that most of them recently happened in the Atlantic salmon branch, while at the same time, *S. trutta* has retained a chromosomal architecture close to the ancestral form of the *Salmo* genus. The reasons for such a contrasted evolution of chromosome structure between these two species remain to be investigated.

### Recombination rate variation across the brown trout genome

Despite extensive chromosomal rearrangements in the *S. salar* lineage, we could identify highly conserved synteny blocks within orthologous chromosome arms to reconstitute local alignments between the brown trout linkage map and the salmon physical map. One of our main objectives was to use these comparisons between maps to estimate local recombination rate variations along each LG in *S. trutta* (Figure 5). The average genome-wide recombination rate was estimated at 0.88 cM/Mb, with strong heterogeneity being found across the genome. Highly recombining regions were observed at the extremities of some LGs (*e.g.*, BT-9; Figure 5), and genomic regions associated with very low recombination rates were also observed (*e.g.*, BT-23; Figure 5). In order to provide an indirect assessment of this local recombination rate estimation for future eco-evolutionary studies, we verified that the frequently observed positive correlation between local recombination rate and nucleotide diversity (Corbett-Detig *et al.* 2015) also occurs in brown trout. In a comparative study across a wide range of species, Corbett-Detig *et al.* (2015) suggested that the strength of this correlation is highly variable among species, being generally stronger in species with large *vs.* small census population size [but see Coop (2016)]. Although brown trout populations are usually considered to have relatively small census sizes (Jorde and Ryman 1996; Palm *et al.* 2003; Jensen *et al.* 2005; Serbezov *et al.* 2012), a significantly positive correlation was found between local recombination rate and nucleotide diversity using polymorphism data from the Atlantic lineage. Thus, recombination rate variation across the brown trout genome shapes the chromosomal landscape of neutral genetic diversity by modulating the efficiency of selection (both positive and negative) at linked neutral sites.

Variation in local recombination rate across genomic regions not only affects genome-wide variation patterns of polymorphism, but also has a profound influence on a range of evolutionary mechanisms of prime importance in brown trout. This recombination map should therefore have beneficial outcomes for our understanding of inbreeding depression, local adaptation, and the historical demography of natural populations (Rezvoy *et al.* 2007; Dukić *et al.* 2016; Racimo *et al.* 2015). It will also be of precious help for understanding genome-wide introgression patterns in natural populations that have been restocked with individuals from a different lineage. Finally, a detailed picture of recombination rate variation in brown trout will help for choosing SNPs for the design of genotyping arrays in future association mapping experiments (*e.g.*, Bourret *et al.* 2013; Houston *et al.* 2014).

### Conclusions

A high-density sex- and lineage-averaged consensus linkage map for *S. trutta* was developed and the morphology (acrocentric or metacentric) of most chromosomes was identified. Interspecies comparisons with the Atlantic salmon allowed identifying genomic regions of highly conserved synteny within chromosome arms and contribute toward a better understanding of recent genome evolution in the genus *Salmo*. A recent and strong acceleration in the rate of chromosomal rearrangements was detected in the *S. salar* branch. These results should stimulate further research toward understanding the contrasted evolution of chromosome structure in sister species with <2% net nucleotide divergence. Recombination rate variation was found to influence genome-wide nucleotide diversity patterns in brown trout. These new genomic resources will provide important tools for future genome scans, QTL mapping, and genome-wide association studies in brown trout. It also paves the way to enable a new generation of conservation genomics approaches that will look at the fitness consequences of introgression of foreign alleles into wild populations at the haplotype level.

## Supplementary Material

Supplemental material is available online at www.g3journal.org/lookup/suppl/doi:10.1534/g3.116.038497/-/DC1.

## References

[bib1] Allendorf F. W. Thorgaard G. H., 1984 Tetraploidy and the evolution of salmonid fishes, pp. 1–53 in *Evolutionary Genetics of Fishes*, *Monographs in Evolutionary Biology*, Volume 1, edited by Turner B. J. Springer, Berlin.

[bib85] Allendorf F. W. Danzmann R. G., 1997 Secondary Tetrasomic Segregation of MDH-B and Preferential Pairing of Homeologues in Rainbow Trout. Genetics 145: 1083–9. 9093860 10.1093/genetics/145.4.1083 PMC1207878

[bib2] Allendorf F. W. Bassham S. Cresko W. A. Limborg M. T. Seeb L. W., 2015 Effects of crossovers between homeologs on inheritance and population genomics in polyploid-derived salmonid fishes. J. Hered. 106: 217–227. 25838153 10.1093/jhered/esv015

[bib3] Almodóvar A. Nicola G. G. Elvira B. García-Marin J. L., 2006 Introgression variability among Iberian brown trout evolutionary significant units: the influence of local management and environmental features. Freshw. Biol. 51: 1175–1187.

[bib4] Amores A. Catchen J. Ferrara A. Fontenot Q. Postlethwait J. H., 2011 Genome evolution and meiotic maps by massively parallel DNA sequencing: spotted gar, an outgroup for the teleost genome duplication. Genetics 188: 799–808. 21828280 10.1534/genetics.111.127324 PMC3176089

[bib5] Bernatchez L., 2001 The evolutionary history of brown trout (*Salmo trutta* L.) inferred from phylogeographic, nested clade, and mismatch analyses of mitochondrial DNA variation. Evolution 55: 351–379. 11308093 10.1111/j.0014-3820.2001.tb01300.x

[bib6] Bernatchez L. Osinov A., 1995 Genetic diversity of trout (genus *Salmo*) from its most eastern native range based on mitochondrial DNA and nuclear gene variation. Mol. Ecol. 4: 285–298. 7663748 10.1111/j.1365-294x.1995.tb00222.x

[bib8] Berrebi P., 2015 Three brown trout *Salmo trutta* lineages in Corsica described through allozyme variation. J. Fish Biol. 86: 60–73. 25353357 10.1111/jfb.12534

[bib9] Berrebi P. Poteaux C. Fissier M. Cattaneo-Berrebi G., 2000 Stocking impact and allozyme diversity in brown trout from Mediterranean southern France. J. Fish Biol. 56: 949–960.

[bib10] Berthelot C. Brunet F. Chalopin D. Juanchich A. Bernard M., 2014 The rainbow trout genome provides novel insights into evolution after whole-genome duplication in vertebrates. Nat. Commun. 5: 3657. 24755649 10.1038/ncomms4657 PMC4071752

[bib11] Bohling J. Haffray P. Berrebi P., 2016 Genetic diversity and population structure of domestic brown trout (*Salmo trutta*) in France. Aquaculture 462: 1–9.

[bib12] Bourret V. Kent M. P. Primmer C. R. Vasemägi A. Karlsson S., 2013 SNP-array reveals genome-wide patterns of geographical and potential adaptive divergence across the natural range of Atlantic salmon (*Salmo salar*). Mol. Ecol. 22: 532–551. 22967111 10.1111/mec.12003

[bib86] Brenna-Hansen S. Li J. Kent M. P. Boulding E. G. Dominik S., 2012 Chromosomal Differences between European and North American Atlantic Salmon Discovered by Linkage Mapping and Supported by Fluorescence in Situ Hybridization Analysis. BMC Genomics 13: 432. 22928605 10.1186/1471-2164-13-432 PMC3495403

[bib13] Brieuc M. S. Waters C. D. Seeb J. E. Naish K. A., 2014 A dense linkage map for Chinook salmon (*Oncorhynchus tshawytscha*) reveals variable chromosomal divergence after an ancestral whole genome duplication event. G3 4: 447–460. 24381192 10.1534/g3.113.009316 PMC3962484

[bib14] Catchen J. Hohenlohe P. A. Bassham S. Amores A. Cresko W. A., 2013 Stacks: an analysis tool set for population genomics. Mol. Ecol. 22: 3124–3140. 23701397 10.1111/mec.12354 PMC3936987

[bib15] Coop G., 2016 Does linked selection explain the narrow range of genetic diversity across species? bioRxiv Available at: http://biorxiv.org/content/early/2016/03/07/042598.

[bib16] Corbett-Detig R. B. Hartl D. L. Sackton T. B., 2015 Natural selection constrains neutral diversity across a wide range of species. PLoS Biol. 13: e1002112. 25859758 10.1371/journal.pbio.1002112 PMC4393120

[bib17] Crête-Lafrenière A. Weir L. K. Bernatchez L., 2012 Framing the Salmonidae family phylogenetic portrait: a more complete picture from increased taxon sampling. PLoS One 7: e46662. 23071608 10.1371/journal.pone.0046662 PMC3465342

[bib18] Dukić M. Berner D. Roesti M. Haag C. R. Ebert D., 2016 A high-density genetic map reveals variation in recombination rate across the genome of *Daphnia magna*. BMC Genet. 17: 137. 27737627 10.1186/s12863-016-0445-7 PMC5064971

[bib19] Ekblom R. Galindo J., 2011 Applications of next generation sequencing in molecular ecology of non-model organisms. Heredity 107: 1–15. 21139633 10.1038/hdy.2010.152 PMC3186121

[bib20] Elliott J. M., 1994 *Quantitative Ecology and the Brown Trout*. Oxford University Press, Oxford.

[bib21] Everett M. V. Miller M. R. Seeb J. E., 2012 Meiotic maps of sockeye salmon derived from massively parallel DNA sequencing. BMC Genomics 13: 521. 23031582 10.1186/1471-2164-13-521 PMC3563581

[bib22] Gagnaire P.-A. Normandeau E. Pavey S. A. Bernatchez L., 2013 a Mapping phenotypic, expression and transmission ratio distortion QTL using RAD markers in the Lake Whitefish (*Coregonus clupeaformis*). Mol. Ecol. 22: 3036–3048. 23181719 10.1111/mec.12127

[bib23] Gagnaire P.-A. Pavey S. A. Normandeau E. Bernatchez L., 2013 b The genetic architecture of reproductive isolation during speciation-with-gene-flow in lake whitefish species pairs assessed by RAD sequencing. Evolution 67: 2483–2497. 24033162 10.1111/evo.12075

[bib24] Gharbi K. Gautier A. Danzmann R. G. Gharbi S. Sakamoto T., 2006 A linkage map for brown trout (*Salmo trutta*): chromosome homeologies and comparative genome organization with other salmonid fish. Genetics 172: 2405–2419. 16452148 10.1534/genetics.105.048330 PMC1456399

[bib25] Gonen S. Lowe N. R. Cezard T. Gharbi K. Bishop S. C., 2014 Linkage maps of the Atlantic salmon (*Salmo salar*) genome derived from RAD sequencing. BMC Genomics 15: 166. 24571138 10.1186/1471-2164-15-166 PMC4028894

[bib26] Hansen M. M. Mensberg K. L. D., 2009 Admixture analysis of stocked brown trout populations using mapped microsatellite DNA markers: indigenous trout persist in introgressed populations. Biol. Lett. 5: 656–659. 19515653 10.1098/rsbl.2009.0214 PMC2781948

[bib27] Hansen M. M. Ruzzante D. E. Nielsen E. E. Mensberg K. D., 2000 Microsatellite and mitochondrial DNA polymorphism reveals life-history dependent interbreeding between hatchery and wild brown trout (*Salmo trutta* L.). Mol. Ecol. 9: 583–594. 10792701 10.1046/j.1365-294x.2000.00898.x

[bib28] Hansen M. M. Meier K. Mensberg K. L. D., 2010 Identifying footprints of selection in stocked brown trout populations: a spatio-temporal approach. Mol. Ecol. 19: 1787–1800. 20345684 10.1111/j.1365-294X.2010.04615.x

[bib29] Hartley S. E. Horne M. T., 1984 Chromosome relationships in the genus Salmo. Chromosoma 90: 229–237. 6499596 10.1007/BF00292401

[bib30] Hecht B. C. Thrower F. P. Hale M. C. Miller M. R. Nichols K. M., 2012 Genetic architecture of migration-related traits in rainbow and steelhead trout, *Oncorhynchus mykiss*. G3 2: 1113–1127. 22973549 10.1534/g3.112.003137 PMC3429926

[bib31] Hohenlohe P. A. Amish S. J. Catchen J. M. Allendorf F. W. Luikart G., 2011 Next-generation RAD sequencing identifies thousands of SNPs for assessing hybridization between rainbow and westslope cutthroat trout. Mol. Ecol. Resour. 11: 117–122. 21429168 10.1111/j.1755-0998.2010.02967.x

[bib32] Houston R. D. Davey J. W. Bishop S. C. Lowe N. R. Mota-Velasco J. C., 2012 Characterisation of QTL-linked and genome-wide restriction site-associated DNA (RAD) markers in farmed Atlantic salmon. BMC Genomics 13: 244. 22702806 10.1186/1471-2164-13-244 PMC3520118

[bib33] Houston R. D. Taggart J. B. Cézard T. Bekaert M. Lowe N. R., 2014 Development and validation of a high density SNP genotyping array for Atlantic salmon (*Salmo salar*). BMC Genomics 15: 90. 24524230 10.1186/1471-2164-15-90 PMC3923896

[bib34] Jensen L. F. Hansen M. M. Carlsson J. Loeschcke V. Mensberg K. L. D., 2005 Spatial and temporal genetic differentiation and effective population size of brown trout (*Salmo trutta*, L.) in small Danish rivers. Conserv. Genet. 6: 615–621.

[bib35] Jorde P. E. Ryman N., 1996 Demographic genetics of brown trout (*Salmo trutta*) and estimation of effective population size from temporal change of allele frequencies. Genetics 143: 1369–1381. 8807308 10.1093/genetics/143.3.1369 PMC1207405

[bib36] Kai W. Nomura K. Fujiwara A. Nakamura Y. Yasuike M., 2014 A ddRAD-based genetic map and its integration with the genome assembly of Japanese eel (*Anguilla japonica*) provides insights into genome evolution after the teleost-specific genome duplication. BMC Genomics 15: 233. 24669946 10.1186/1471-2164-15-233 PMC3986909

[bib37] Kodama M. Brieuc M. S. O. Devlin R. H. Hard J. J. Naish K. A., 2014 Comparative mapping between coho salmon (*Oncorhynchus kisutch*) and three other salmonids suggests a role for chromosomal rearrangements in the retention of duplicated regions following a whole genome duplication event. G3 4: 1717–1730. 25053705 10.1534/g3.114.012294 PMC4169165

[bib38] Kuang Y.-Y. Zheng X.-H. Li C.-Y. Li X.-M. Cao D.-C., 2016 The genetic map of goldfish (*Carassius auratus*) provided insights to the divergent genome evolutions in the Cyprinidae family. Sci. Rep. 6: 34849. 27708388 10.1038/srep34849 PMC5052598

[bib39] Kumar G. Kocour M., 2017 Applications of next-generation sequencing in fisheries research: a review. Fish. Res. 186: 11–22.

[bib87] Laikre L., 1999 Conservation genetic management of brown trout (Salmo trutta) in Europe.

[bib40] Lamaze F. C. Sauvage C. Marie A. Garant D. Bernatchez L., 2012 Dynamics of introgressive hybridization assessed by SNP population genomics of coding genes in stocked brook charr (*Salvelinus fontinalis*). Mol. Ecol. 21: 2877–2895. 22548328 10.1111/j.1365-294X.2012.05579.x

[bib41] Largiader C. R. Scholl A., 1996 Genetic introgression between native and introduced brown trout *Salmo trutta* L. populations in the Rhone River Basin. Mol. Ecol. 5: 417–426.

[bib42] Larson W. A. Seeb L. W. Everett M. V. Waples R. K. Templin W. D., 2014 Genotyping by sequencing resolves shallow population structure to inform conservation of Chinook salmon (*Oncorhynchus tshawytscha*). Evol. Appl. 7: 355–369. 24665338 10.1111/eva.12128 PMC3962296

[bib43] Larson W. A. McKinney G. J. Limborg M. T. Everett M. V. Seeb L. W., 2016 Identification of multiple QTL hotspots in sockeye salmon (*Oncorhynchus nerka*) using genotyping-by-sequencing and a dense linkage map. J. Hered. 107: 122–133. 26712859 10.1093/jhered/esv099 PMC5994969

[bib44] Leitwein M. Gagnaire P. A. Desmarais E. Guendouz S. Rohmer M., 2016 Genome-wide nucleotide diversity of hatchery-reared Atlantic and Mediterranean strains of brown trout *Salmo trutta* compared to wild Mediterranean populations. J. Fish Biol. 89: 2717–2734. 27666575 10.1111/jfb.13131

[bib45] Li H. Durbin R., 2010 Fast and accurate long-read alignment with Burrows–Wheeler transform. Bioinformatics 26: 589–595. 20080505 10.1093/bioinformatics/btp698 PMC2828108

[bib88] Li H., 2011 Statistical Framework for SNP Calling, Mutation Discovery, Association Mapping and Population Genetical Parameter Estimation from Sequencing Data. Bioinformatics 27: 2987–93. 21903627 10.1093/bioinformatics/btr509 PMC3198575

[bib89] Lien S. Gidskehaug L. Moen T. Hayes B. J. Berg P. R., 2011 A Dense SNP-Based Linkage Map for Atlantic Salmon (Salmo Salar) Reveals Extended Chromosome Homeologies and Striking Differences in Sex-Specific Recombination Patterns. BMC Genomics 12: 615. 22182215 10.1186/1471-2164-12-615 PMC3261913

[bib46] Lien S. Koop B. F. Sandve S. R. Miller J. R. Kent M. P., 2016 The Atlantic salmon genome provides insights into rediploidization. Nature 533: 200–205. 27088604 10.1038/nature17164 PMC8127823

[bib47] Limborg M. T. Waples R. K. Seeb J. E. Seeb L. W., 2014 Temporally isolated lineages of pink salmon reveal unique signatures of selection on distinct pools of standing genetic variation. J. Hered. 105: 835–845. 10.1093/jhered/esu063 25292170

[bib48] Limborg M. T. McKinney G. J. Seeb L. W. Seeb J. E., 2015 a Recombination patterns reveal information about centromere location on linkage maps. Mol. Ecol. Resour. 16: 655–661. 26561199 10.1111/1755-0998.12484

[bib49] Limborg M. T. Waples R. K. Allendorf F. W. Seeb J. E., 2015 b Linkage mapping reveals strong chiasma interference in sockeye salmon: implications for interpreting genomic data. G3 5: 2463–2473. 26384769 10.1534/g3.115.020222 PMC4632065

[bib50] Liu S. Li Y. Qin Z. Geng X. Bao L., 2016 High-density interspecific genetic linkage mapping provides insights into genomic incompatibility between channel catfish and blue catfish. Anim. Genet. 47: 81–90. 26537786 10.1111/age.12372

[bib51] Macqueen D. J. Johnston I. A., 2014 A well-constrained estimate for the timing of the salmonid whole genome duplication reveals major decoupling from species diversification. Proc. R. Soc. Lond. B Biol. Sci. 281: 20132881. 10.1098/rspb.2013.2881 PMC3906940 24452024

[bib52] Martinez P. Vinas A. Bouza C. Arias J. Amaro R., 1991 Cytogenetical characterization of hatchery stocks and natural populations of sea and brown trout from northwestern Spain. Heredity 66: 9–17.

[bib53] May B. Delany M. E., 2015 Meiotic models to explain classical linkage, pseudolinkage, and chromosomal pairing in tetraploid derivative salmonid genomes: II. Wright is still right. J. Hered. 106: 762–766. 26320244 10.1093/jhered/esv056 PMC4642675

[bib54] McKinney G. L. Seeb W. Larson D. Gomez-Uchida M. Limborg M., 2015 An integrated linkage map reveals candidate genes underlying adaptive variation in Chinook salmon (*Oncorhynchus tshawytscha*). Mol. Ecol. Resour. 16: 769–783. 26490135 10.1111/1755-0998.12479

[bib55] McMeel O. M. Hoey E. M. Ferguson A., 2001 Partial nucleotide sequences, and routine typing by polymerase chain reaction–restriction fragment length polymorphism, of the brown trout (*Salmo trutta*) lactate dehydrogenase, LDH-C1*90 and *100 alleles. Mol. Ecol. 10: 29–34. 11251784 10.1046/j.1365-294x.2001.01166.x

[bib56] Morrison W. J., 1970 Nonrandom segregation of two lactate dehydrogenase subunit loci in trout. Trans. Am. Fish. Soc. 99: 193–206.

[bib57] Near T. J. Eytan R. I. Dornburg A. Kuhn K. L. Moore J. A., 2012 Resolution of ray-finned fish phylogeny and timing of diversification. Proc. Natl. Acad. Sci. USA 109: 13698–13703. 22869754 10.1073/pnas.1206625109 PMC3427055

[bib58] Nugent C. M. Easton A. A. Norman J. D. Ferguson M. M. Danzmann R. G., 2017 A SNP based linkage map of the Arctic charr (*Salvelinus alpinus*) genome provides insights into the diploidization process after whole genome duplication. G3 7: 543–556. 27986793 10.1534/g3.116.038026 PMC5295600

[bib59] Ohno S., 1970 The enormous diversity in genome sizes of fish as a reflection of nature’s extensive experiments with gene duplication. Trans. Am. Fish. Soc. 99: 120–130.

[bib60] Palm S. Laikre L. Jorde P. E. Ryman N., 2003 Effective population size and temporal genetic change in stream resident brown trout (*Salmo trutta*, *L*.). Conserv. Genet. 4: 249–264.

[bib61] Perrier C. Bourret V. Kent M. P. Bernatchez L., 2013 Parallel and nonparallel genome-wide divergence among replicate population pairs of freshwater and anadromous Atlantic salmon. Mol. Ecol. 22: 5577–5593. 24730037 10.1111/mec.12500

[bib62] Peterson B. K. Weber J. N. Kay E. H. Fisher H. S. Hoekstra H. E., 2012 Double digest RADseq: an inexpensive method for *de novo* SNP discovery and genotyping in model and non-model species. PLoS One 7: e37135. 22675423 10.1371/journal.pone.0037135 PMC3365034

[bib63] Phillips R. Ráb P., 2001 Chromosome evolution in the Salmonidae (Pisces): an update. Biol. Rev. Camb. Philos. Soc. 76: 1–25. 11325050 10.1017/s1464793100005613

[bib65] Poteaux C. Beaudou D. Berrebi P., 1998 Temporal variations of genetic introgression in stocked brown trout populations. J. Fish Biol. 53: 701–713.

[bib66] Poteaux C. Bonhomme F. Berrebi P., 1999 Microsatellite polymorphism and genetic impact of restocking in Mediterranean brown trout (*Salmo trutta L*.). Heredity 82: 645–653. 10383686 10.1046/j.1365-2540.1999.00519.x

[bib67] Racimo F. Sankararaman S. Nielsen R. Huerta-Sánchez E., 2015 Evidence for archaic adaptive introgression in humans. Nat. Rev. Genet. 16: 359–371. 25963373 10.1038/nrg3936 PMC4478293

[bib68] Rezvoy C. Charif D. Guéguen L. Marais G. A., 2007 MareyMap: an R-based tool with graphical interface for estimating recombination rates. Bioinformatics 23: 2188–2189. 17586550 10.1093/bioinformatics/btm315

[bib69] Rondeau E. B. Minkley D. R. Leong J. S. Messmer A. M. Jantzen J. R., 2014 The genome and linkage map of the northern pike (*Esox lucius*): conserved synteny revealed between the salmonid sister group and the Neoteleostei. PLoS One 9: e102089. 25069045 10.1371/journal.pone.0102089 PMC4113312

[bib70] Sanz N. Cortey M. Pla C. García-Marín J. L., 2006 Hatchery introgression blurs ancient hybridization between brown trout (*Salmo trutta*) lineages as indicated by complementary allozymes and mtDNA markers. Biol. Conserv. 130: 278–289.

[bib71] Sauvage C. Vagner M. Derôme N. Audet C. Bernatchez L., 2012 Coding gene single nucleotide polymorphism mapping and quantitative trait loci detection for physiological reproductive traits in brook charr, *Salvelinus fontinalis*. G3 2: 379–392. 22413092 10.1534/g3.111.001867 PMC3291508

[bib72] Serbezov D. Jorde P. E. Bernatchez L. Olsen E. M. Vøllestad L. A., 2012 Life history and demographic determinants of effective/census size ratios as exemplified by brown trout (*Salmo trutta*). Evol. Appl. 5: 607–618. 23028401 10.1111/j.1752-4571.2012.00239.x PMC3461143

[bib73] Slate J. Gratten J. Beraldi D. Stapley J. Hale M., 2008 Gene mapping in the wild with SNPs: guidelines and future directions. Genetica 136: 97–107. 18780148 10.1007/s10709-008-9317-z

[bib74] Stapley J. Reger J. Feulner P. G. D. Smadja C. Galindo J., 2010 Adaptation genomics: the next generation. Trends Ecol. Evol. 25: 705–712. 20952088 10.1016/j.tree.2010.09.002

[bib75] Sutherland B. J. G. Gosselin T. Normandeau E. Lamothe M. Isabel N., 2016 Salmonid chromosome evolution as revealed by a novel method for comparing RADseq linkage maps. Genome Biol. Evol. 8: 3600–3617. 28173098 10.1093/gbe/evw262 PMC5381510

[bib76] Thaulow J. Borgstrøm R. Heun M., 2014 Genetic persistence of an initially introduced brown trout (*Salmo trutta L*.) population despite restocking of foreign conspecifics. Ecol. Freshwat. Fish 23: 485–497.

[bib77] Tsai H. Y. Hamilton A. Guy D. R. Tinch A. E. Bishop S. C., 2015 The genetic architecture of growth and fillet traits in farmed Atlantic salmon (*Salmo salar*). BMC Genet. 16: 51. 25985885 10.1186/s12863-015-0215-y PMC4436873

[bib78] Tsai H. Y. Robledo D. Lowe N. R. Bekaert M. Taggart J. B., 2016 Construction and annotation of a high density SNP linkage map of the Atlantic salmon (*Salmo salar*) genome. G3 6: 2173–2179. 27194803 10.1534/g3.116.029009 PMC4938670

[bib79] Van Ooijen J. W., 2006 *JoinMap 4*; *Software for the Calculation of Genetic Map in Experimental Populations*. Kyazma B.V., Wageningen, Netherlands.

[bib80] Wang L. Wan Z. Y. Bai B. Huang S. Q. Chua E., 2015 Construction of a high-density linkage map and fine mapping of QTL for growth in Asian seabass. Sci. Rep. 5: 16358. 26553309 10.1038/srep16358 PMC4639833

[bib81] Waples R. K. Seeb L. W. Seeb J. E., 2016 Linkage mapping with paralogs exposes regions of residual tetrasomic inheritance in chum salmon (*Oncorhynchus keta*). Mol. Ecol. Resour. 16: 17–28. 25712438 10.1111/1755-0998.12394

[bib82] Wright J. E. Jr Johnson K. Hollister A. May B., 1983 Meiotic models to explain classical linkage, pseudolinkage, and chromosome pairing in tetraploid derivative salmonid genomes. Isozymes 10: 239–260. 6354984

[bib83] Yano A. Nicol B. Jouanno E. Quillet E. Fostier A., 2013 The sexually dimorphic on the Y-chromosome gene (sdY) is a conserved male-specific Y-chromosome sequence in many salmonids. Evol. Appl. 6: 486–496. 23745140 10.1111/eva.12032 PMC3673476

[bib84] Zhao L. Zhang Y. Ji P. Zhang X. Zhao Z., 2013 A dense genetic linkage map for common carp and its integration with a BAC-based physical map. PLoS One 8: e63928. 23704958 10.1371/journal.pone.0063928 PMC3660343

