# Supplementary figures and images for: A Dense Brown Trout (Salmo trutta) Linkage Map Reveals Recent Chromosomal Rearrangements in the Salmo Genus and the Impact of Selection on Linked Neutral Diversity
Source: G3 (Bethesda). 2017 Feb 24;7(4):1365–76. doi: 10.1534/g3.116.038497 (PMC5386884; doi:10.1534/g3.116.038497)

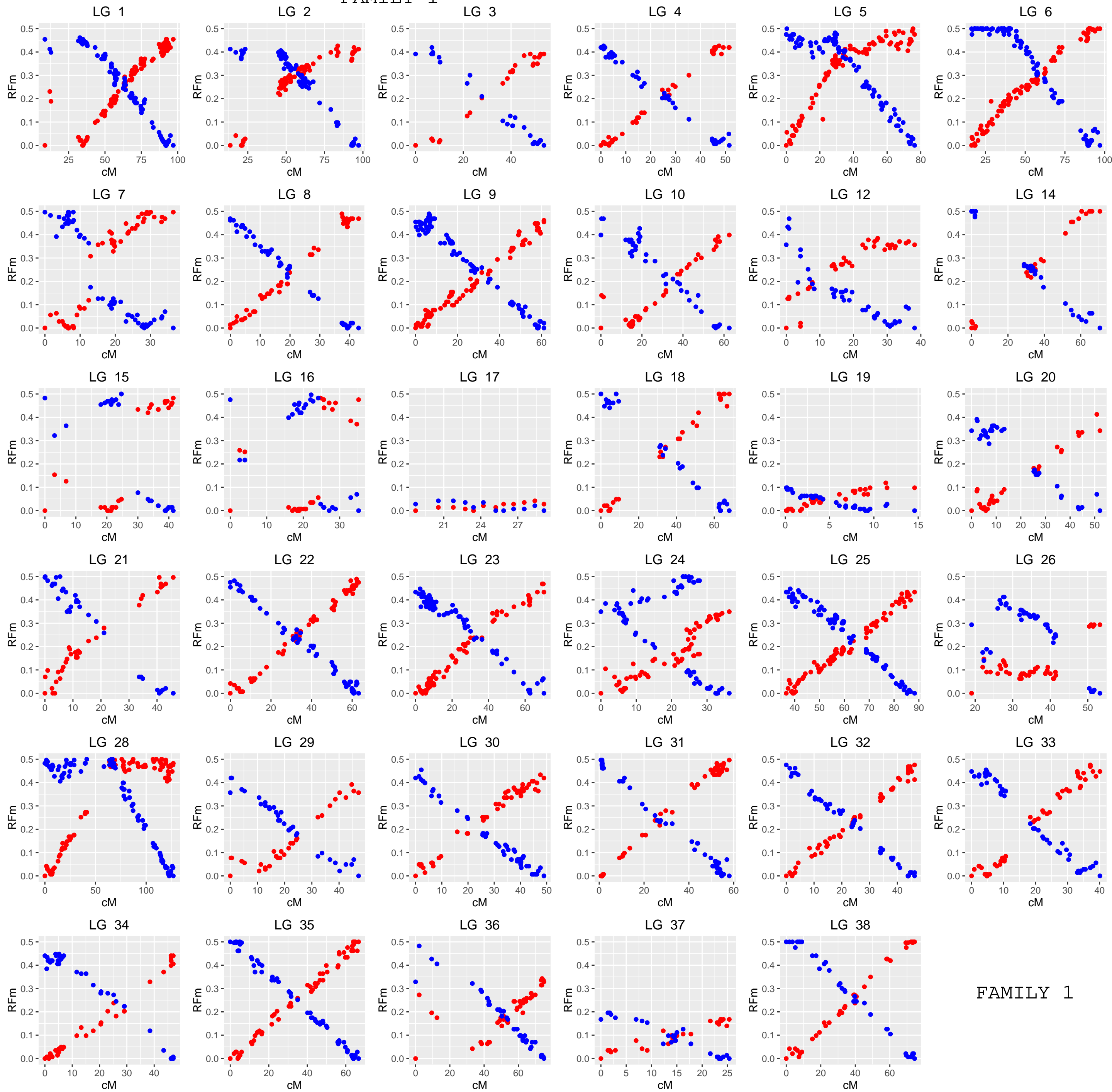

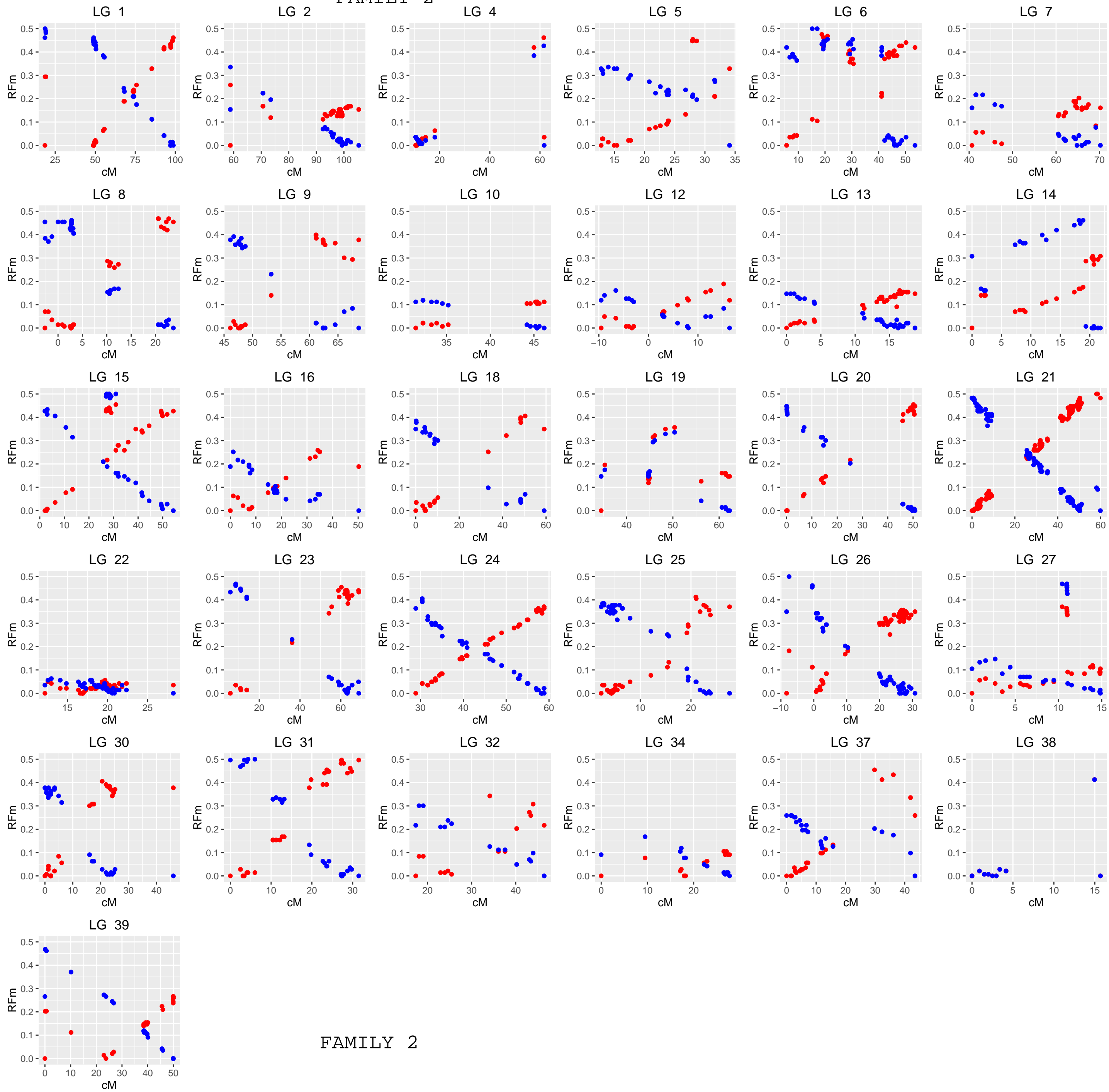

Supplement: Supplementary file 2 [file 1365FigureS1.pdf]

Map comparison for species trutta and salar

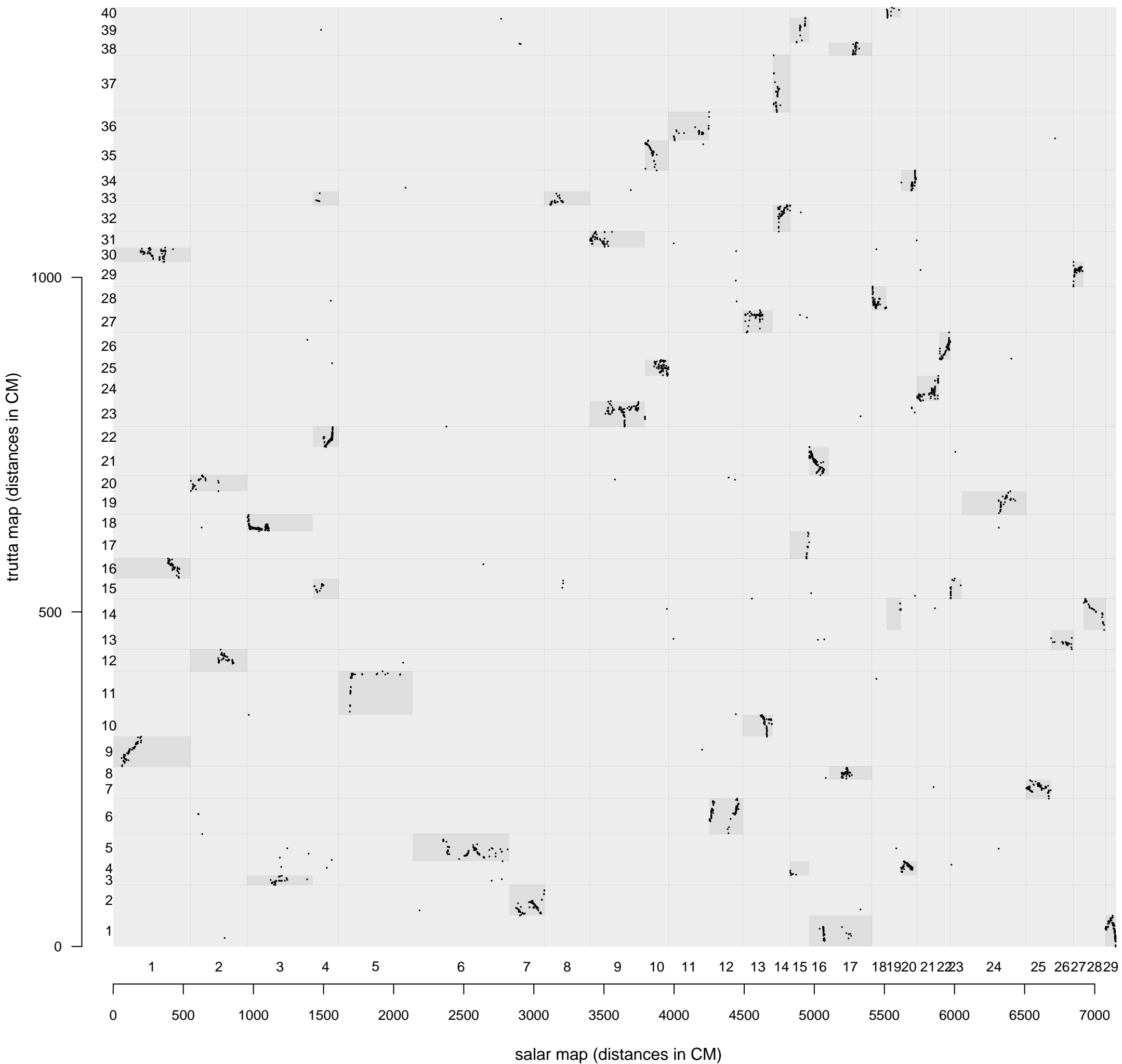

Supplement: Supplementary file 3 [file 1365FigureS2.pdf]

# Position of male and female markers along each LG

## Family 1

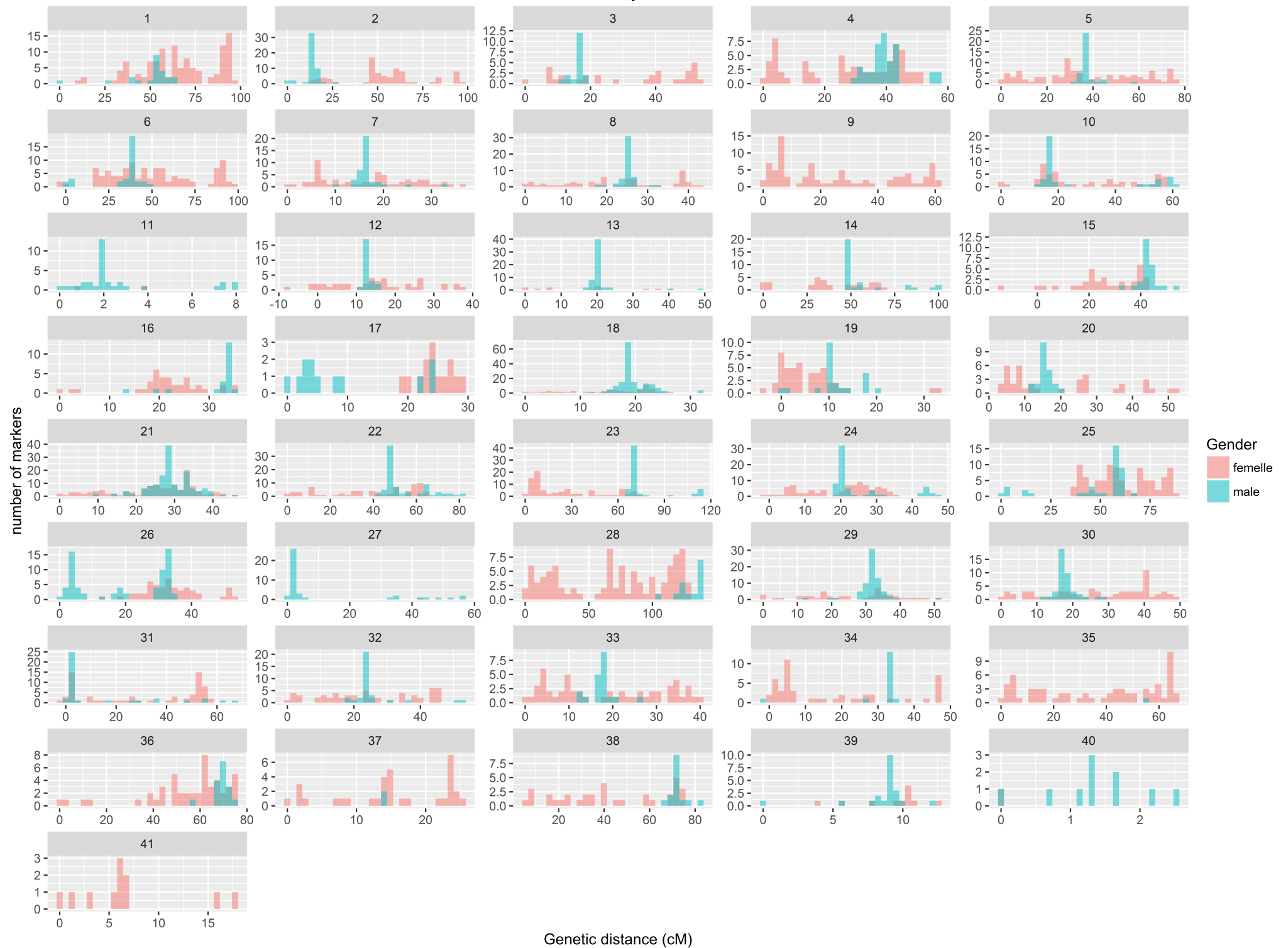

Supplement: Supplementary file 4 [file 1365FigureS3.pdf]
