# Supplementary material for: A Dense Brown Trout (Salmo trutta) Linkage Map Reveals Recent Chromosomal Rearrangements in the Salmo Genus and the Impact of Selection on Linked Neutral Diversity
Source: G3 (Bethesda). 2017 Feb 24;7(4):1365–76. doi: 10.1534/g3.116.038497 (PMC5386884; doi:10.1534/g3.116.038497)
Supplement: Supplementary file 6 [file 1365TableS2.docx]

| markers | LG_Map Gharbi | Locus RAD | LG_Map RAD | Position RAD(cM) | Markers | LG_Map Gharbi | Locus RAD | LG_Map RAD | | Position_RAD(cM) |
| --- | --- | --- | --- | --- | --- | --- | --- | --- | --- | --- |
| *Ssa23NVH* | 1 | 219155 | 6 | 27.864 | *Ssa39NVH* | 22 | 355668 | 19 | 22.472 | |
| *Ssa77NUIG* | 1 | 209389 | 6 | 35.896 | *Ssa7NVH* | 22 | 350656 | 19 | 26.975 | |
| *MST542* | 2 | 166733 | 31 | 2.246 | *OMM1152* | 23 | 287286 | 28 | 8.529 | |
| *Ssa35NVH* | 2 | 173958 | 23 | 24.754 | *Omy9INRA* | 23 | 80181 | 30 | 15.572 | |
| *SSOSL32* | 2 | 158825 | 23 | 21.728 | *Ssa21NVH* | 23 | 287763 | 28 | 17.285 | |
| *OmyRGT36TUF* | 4 | 343726 | 15 | 29.805 | *Ssa42NVH* | 23 | 295313 | 28 | 2.453 | |
| *Oneµ2* | 4 | 346006 | 15 | 16.945 | *Ssa24NVH* | 24 | 101848 | 3 | 6.462 | |
| *Ssa85* | 4 | 345535 | 15 | 19.576 | *Ssa6NVH* | 25 | 273441 | 21 | 6.974 | |
| *Omy14INRA* | 5 | 144253 | 2 | 13.866 | *OMM1029* | 26 | 227610 | 27 | 0.695 | |
| *Ocl8* | 6 | 387283 | 1 | 12.235 | *Ssa86NVH* | 26 | 236246 | 27 | 30.987 | |
| *Ssa64NVH* | 6 | 298181 | 5 | 19.18 | *SSOSL439* | 26 | 224963 | 27 | 26.128 | |
| *Omy1INRA* | 7 | 177511 | 25 | 0.977 | *Strutta58* | 26 | 223978 | 27 | 25.283 | |
| *Ssa158NVH* | 7 | 190542 | 25 | 11.695 | *OMM1130* | 27 | 232051 | 10 | 18.173 | |
| *Ssa28NVH* | 7 | 190439 | 25 | 4.885 | *Ots107* | 27 | 215909 | 6 | 35.401 | |
| *Ssa156NVH* | 9 | 358531 | 7 | 21.142 | *Ssa90NVH* | 27 | 236532 | 10 | 4.801 | |
| *MST60* | 10 | 276720 | 1 | 10.062 | *Omy325UoG* | 28 | 265036 | 17 | 17.958 | |
| *Omy77* | 10 | 275878 | 1 | 12.001 | *OmyRT5TUF* | 28 | 258863 | 39 | 35.37 | |
| *c417* | 12 | 180790 | 35 | 28.116 | *Ssa197* | 28 | 265426 | 17 | 17.313 | |
| *OMM1070* | 12 | 125555 | 11 | 60.387 | *Ssa11NVH* | 29 | 375077 | 29 | 16.715 | |
| *OMM1108* | 12 | 126996 | 11 | 60.566 | *Ssa62NVH* | 29 | 377098 | 29 | 26.561 | |
| *OmyFGT5TUF* | 12 | 123609 | 11 | 61.15 | *Str2INRA* | 29 | 375267 | 29 | 15.833 | |
| *Ssa38NVH* | 12 | 119193 | 11 | 56.411 | *OMM1107* | 30 | 117285 | 22 | 14.841 | |
| *Ssa40NVH* | 12 | 121895 | 11 | 60.61 | *Ssa159NVH* | 30 | 115200 | 22 | 13.582 | |
| *Ssa161NVH* | 13 | 201724 | 36 | 8.804 | *SSLEEN82* | 30 | 112534 | 22 | 8.846 | |
| *Ssa162NVH* | 13 | 196581 | 36 | 5.312 | *One107* | 31 | 323353 | 24 | 10.383 | |
| *Ssa20NVH* | 13 | 199676 | 9 | 25.181 | *Ssa100NVH* | 31 | 324601 | 24 | 12.466 | |
| *Ssa408UoS* | 13 | 203047 | 36 | 14.481 | *Ssa84NVH* | 31 | 330272 | 24 | 33.892 | |
| *SL* | 14 | 319749 | 34 | 8.271 | *Oki10* | 32 | 314470 | 4 | 8.911 | |
| *Ssa140NVH* | 14 | 318635 | 34 | 9.103 | *Ssa103NVH* | 32 | 310856 | 4 | 17.902 | |
| *Ogo5* | 15 | 282327 | 38 | 2.473 | *Ssa4DIAS* | 32 | 313972 | 4 | 10.592 | |
| *Sfo4* | 15 | 285010 | 2 | 9.119 | *Ssa179NVH* | 33 | 334773 | 26 | 19.435 | |
| *Ocl4* | 16 | 102679 | 3 | 13.732 | *Ssa419UoS* | 33 | 340041 | 26 | 14.999 | |
| *Ssa121NVH* | 16 | 95842 | 18 | 3.573 | *Ogo8* | 35 | 183981 | 35 | 13.823 | |
| *Ssa417UoS* | 16 | 98640 | 18 | 10.254 | *Ssa63NVH* | 35 | 178445 | 35 | 34.207 | |
| *Ssa41NVH* | 16 | 97063 | 18 | 5.201 | *SSOSL417* | 35 | 141346 | 5 | 1.945 | |
| *Ssa89NVH* | 16 | 96704 | 18 | 1.724 | *OMM1090* | 36 | 381784 | 14 | 11.194 | |
| *MST543* | 17 | 171677 | 31 | 5.686 | *Ssa207NVH* | 37 | 237401 | 32 | 28.71 | |
| *Ssa5NVH* | 17 | 163022 | 31 | 12.923 | *MST591** | x | 175715 | 25 | 11.1 | |
| *OMM1105* | 18 | 136935 | 5 | 12.831 | *MST85** | x | 171551 | 23 | 25.748 | |
| *OMM1161* | 18 | 132208 | 5 | 18.821 | *omy21DIAS** | x | 128654 | 12 | 12.942 | |
| *Omy4DIAS* | 18 | 136270 | 5 | 9.546 | *ssosl311** | x | 178791 | 35 | 30.827 | |
| *OmyRGT10TUF* | 19 | 256891 | 39 | 26.518 | *StrBS131R** | x | 266043 | 21 | 24.396 | |
| *Oneµ9* | 19 | 257253 | 39 | 29.502 | *Ldhxon3F*** | x | 271508 | 21 | 31.708 | |
| *Ssa12NVH* | 19 | 256380 | 12 | 13.572 |  |  |  |  |  | |
| *Ocl9* | 22 | 356079 | 19 | 23.066 |  |  |  |  |  | |

**Table 2 :** Position of the microsatellites markers on LGs of the low density linkage map provided in Gharbi et al. (2006) (LG_Map Gharbi) and their correspondence on the LGs of the *Salmo trutta* high density linkage map developed in this study (LG_Map RAD). The position (in CM) for each of these markers is also provided (Position RAD (cM)). The position of five microsatellite markers used in Leitwein et al. (2016), and one LDH marker used in (Mc Meel et al. 2001) are also provided at the end of the right column. These markers were not considered in Gharbi et al. (2006) and their positions on the low density linkage map are not defined (X).

*microsatellites used in Leitwein et al. (2016) and not found on Gharbi et al. (2006) linkage map

**LDH marker
